# Epidemiology, Mechanisms of Resistance and Treatment Algorithm for Infections Due to Carbapenem-Resistant Gram-Negative Bacteria: An Expert Panel Opinion

**DOI:** 10.3390/antibiotics11091263

**Published:** 2022-09-17

**Authors:** Nicola Coppola, Alberto Enrico Maraolo, Lorenzo Onorato, Riccardo Scotto, Federica Calò, Luigi Atripaldi, Anna Borrelli, Antonio Corcione, Maria Giovanna De Cristofaro, Emanuele Durante-Mangoni, Amelia Filippelli, Gianluigi Franci, Maria Galdo, Gaspare Guglielmi, Pasquale Pagliano, Alessandro Perrella, Ornella Piazza, Marco Picardi, Rodolfo Punzi, Ugo Trama, Ivan Gentile

**Affiliations:** 1 Infectious Diseases Unit, Department of Mental Health and Public Medicine, University of Campania Luigi Vanvitelli, 80138 Naples, Italy; 2 Emerging Infectious Disease with High Contagiousness Unit, Cotugno Hospital, AORN Dei Colli, 80131 Naples, Italy; 3 Infectious Diseases Unit, Department of Clinical Medicine and Surgery, University of Naples Federico II, 80138 Naples, Italy; 4 Clinical Pathology Unit, Cotugno Hospital, AORN Dei Colli, 80131 Naples, Italy; 5 Direzione Sanitaria, “San Giovanni di Dio e Ruggi d’Aragona” University Hospital, 84125 Salerno, Italy; 6 Intensive Care Unit, Monaldi Hospital, AORN Dei Colli, 80131 Naples, Italy; 7 Intensive Care Unit, AORN Cardarelli, 80131 Naples, Italy; 8 Department of Precision Medicine, University of Campania ‘L. Vanvitelli’ and Unit of Infectious and Transplant Medicine, Monaldi Hospital, AORN Ospedali dei Colli, 80131 Naples, Italy; 9 Department of Medicine Surgery and Dentistry, University of Salerno and Clinical Pharmacology and Pharmacogenetics Unit, “San Giovanni di Dio e Ruggi d’Aragona” University Hospital, 84125 Salerno, Italy; 10 Department of Medicine Surgery and Dentistry, University of Salerno and Clinical Pathology and Microbiology Unit, “San Giovanni di Dio e Ruggi D’Aragona” University Hospital, 84125 Salerno, Italy; 11 Pharmacy Unit, AORN Dei Colli, 80131 Naples, Italy; 12 Pharmacy Unit, AORN Cardarelli, 80131 Naples, Italy; 13 Department of Medicine Surgery and Dentistry, University of Salerno, Infectious Diseases Unit, 84125 Salerno, Italy; 14 Department of Medicine, Surgery and Dentistry, University of Salerno, Unit of Anesthesiology, 84125 Salerno, Italy; 15 Department of Clinical Medicine and Surgery, Hematology Unit, Federico II University, 80131 Naples, Italy; 16 Hepatic Infectious Disease Unit, Cotugno Hospital, AORN Dei Colli, 80131 Naples, Italy; 17 UOSD Politica del Farmaco e Dispositivi, Campania region, 80143 Naples, Italy

**Keywords:** carbapenem-resistant organisms, antimicrobial resistance, *Enterobacterales*, *Acinetobacter baumannii*, *Pseudomonas aeruginosa*, *Stenotrophomonas maltophilia*

## Abstract

Antimicrobial resistance represents a serious threat for global health, causing an unacceptable burden in terms of morbidity, mortality and healthcare costs. In particular, in 2017, carbapenem-resistant organisms were listed by the WHO among the group of pathogens for which novel treatment strategies are urgently needed. Fortunately, several drugs and combinations have been introduced in recent years to treat multi-drug-resistant (MDR) bacteria. However, a correct use of these molecules is needed to preserve their efficacy. In the present paper, we will provide an overview on the epidemiology and mechanisms of resistance of the most common MDR Gram-negative bacteria, proposing a treatment algorithm for the management of infections due to carbapenem-resistant bacteria based on the most recent clinical evidence.

## 1. Introduction

Despite the global shift in attention toward the SARS-CoV-2 infection, antimicrobial resistance (AMR) still represents a serious threat and it should be considered as a silent undervalued pandemic. The rate of multi-drug-resistant (MDR) strains of Gram-negative bacteria is of great concern across the Europe, with particular attention to *Klebsiella pneumoniae* and *Acinetobacter* spp. [1]. According to the latest European Centre for Disease Prevention and Control (ECDC) report on antimicrobial resistance in Europe, referring to 2020 data, *K. pneumoniae* strains showed a very high rate of resistance to carbapenems (>25%) in about one-third of European countries, while the prevalence of MDR *Acinetobacter* spp. was even higher, with rates of strains resistant to carbapenems of >50% in more than half of the European countries [1]. The epidemiological situation of other MDR gram-negative bacteria was not acceptable, also considering that each year there are more than 670,000 infections due to antibiotic-resistant bacteria in Europe, with more than 30,000 people dying as direct consequence of these infections [2]. It is also becoming clear that the SARS-CoV-2 pandemic has had a negative impact on the implementation of Antimicrobial Stewardship Programs (ASP) and on the prevalence of MDR strains in countries particularly engaged in fighting COVID-19 [3]. In this scenario, an increase in the rate of MDR isolates, including Gram-negative strains, is expected. Fortunately, several drugs and combinations have been introduced in recent years to treat MDR bacteria. However, an appropriate and conscientious use of these drugs is needed to preserve their long-lasting efficacy. In this perspective, the five Ds of antimicrobial stewardship must always be applied when treating infections caused by MDR bacteria in the clinical setting (Table 1).

With the present paper, we aim to develop a therapeutic algorithm for the treatment of infections caused by gram-negative MDR bacteria, conceived in accordance with international literature and the principles of conscientious use of antibiotics. In this perspective, we evaluated the mechanisms of resistance of Gram-negative bacteria and the more recent drugs and combinations effective against these pathogens.

## 2. Methods

In 2021, a panel of 17 experts of antimicrobial therapy operating in the Campania region, in Southern Italy, worked to develop an algorithm for the empirical and targeted treatment of infections due to carbapenem-resistant Gram-negative bacteria (GNB) in order to provide to the prescribers a guidance for the management of these difficult-to-treat infections. Moreover, NC, RS, LO, AM and IG performed an extensive review of the available evidence, using published material in the English language, and submitted to all panel members the results of their work. After a discussion, a consensus was reached, and the proposed algorithm was sent for evaluation to all the panel, that finally approved it. In the following paragraphs, we will describe the algorithm developed for the targeted treatment.

### 2.1. Epidemiology of Carbapenem-Resistant Pathogens and Mechanisms of Resistance

#### Carbapenem-Resistant Enterobacterales (CRE)

In 2019, the European Centre for Disease Prevention and Control (ECDC) documented an increasing prevalence of carbapenem-resistant Enterobacterales (CRE) in Europe, with 43% of countries reporting regional or inter-regional spread of CRE [4]. In 2017, a total of 137,728 invasive isolate of carbapenem-resistant *E. coli* and 32,461 invasive isolates of carbapenem-resistant *K. pneumoniae* were reported from European countries [5].

Several mechanisms are responsible for carbapenem resistance among Enterobacterales (i.e., alterations of the penicillin-binding proteins, decrease in bacterial membrane permeability and increase in efflux pumps), although the production of carbapenemase enzymes is the most represented mechanism of resistance, with 85% of CRE worldwide producing carbapenemases [6,7]. According to the Amber classification of ß-lactamases, carbapenemases are found in Ambler’s class A, B and D (Table 2). Molecular identification of the specific carbapenemase is critical for the choice of appropriate antibiotic [8].

### 2.2. Pseudomonas aeruginosa with Difficult-to-Treat Resistance (DTR-PA)

*P. aeruginosa* is the most common cause of infection among non-fermenting Gram-negative bacteria, frequently involved in nosocomial setting and affecting fragile populations [9].

Carbapenem-resistant *P. aeruginosa* (CRPA) isolates represent a global health menace, similarly to Enterobacterales species as well as *A. baumannii*: they all are considered “top priority pathogens” for which novel antibiotics are urgently, needed according to the World Health Organization [10].

DTR-*P. aeruginosa* (PA) is defined by the non-susceptibility to all of the following first-line agents among beta-lactams and quinolones: ceftazidime, cefepime, piperacillin-tazobactam, aztreonam, ciprofloxacin, levofloxacin, meropenem and imipenem-cilastatin [11]. DTR-PA, as far as *P. aeruginosa* was concerned, represented the focus of the guideline for Gram-negative infections by resistant pathogens endorsed by the Infectious Diseases Society of America (IDSA) [12]. Subsequent European guidelines, endorsed by both the European Society of Clinical Microbiology and Infectious Diseases (ESCMID) and the European Society of Intensive Care Medicine (ESICM), referred to DTR-CRPA [13]. Eventually, the very recent Italian guidelines addressing infections by MDR bacteria also focused on DTR-PA [14].

### 2.3. Carbapenem-Resistant A. baumannii (CRAB)

*A. baumannii* is a Gram-negative, strictly aerobic, non-fermentative coccobacillus; it can be present in humans as a colonizer (in the skin, in the respiratory tract and in the digestive system) and can be isolated in the healthcare environment due to its ability to produce robust biofilms. It has emerged as a major cause of health-care-associated infections and, in subjects at risk, it behaves as an opportunistic pathogen causing wound infections, urinary tract and respiratory tract infections including community-acquired and, most frequently, hospital-acquired or ventilator-associated pneumonia [15]. It is considered a difficult-to-treat bug due to its intrinsic and acquired resistance to various classes of antibiotics, with a more worrying increasing resistance to carbapenems and colistin with some geographic variations in the resistance patterns.

Indeed, the percentages of carbapenem-resistant *Acinetobacter* spp. are equal to or above 50% in 21 (55%) countries/areas in southern and eastern Europe [1].

Common mechanisms of resistance include enzymatic inactivation by beta-lactamases, overexpression of drug efflux pumps, and mutations in antibiotic binding targets [16,17].

Resistance to carbapenems is mainly mediated by oxacillinases. The blaOXA-51 gene is an intrinsic gene of *A. baumannii* species, while other oxacillinases are plasmid-acquired such as those of group 23 (includes OXA-27 and OXA-49), (24)-40-like (includes OXA-25, OXA-26, and OXA-40), 58, 143 and 235 [18,19,20]. *Acinetobacter* more rarely hosts metallo-enzymes, mostly represented by Verona Integron-encoded metallo-beta-lactamase (VIM) and Imipenemase (IMP) and less frequently New Delhi metallo-beta-lactamase-1 (NDM-1) and 2.

Besides resistance to the beta-lactam group of antimicrobials, resistance to other classes of antibiotics is almost always present in the *Acinetobacter* species. Aminoglycoside resistance is mediated by plasmid- or transposon-coded Aminoglycoside-Modifying Enzymes (AMEs) [21]. Reduced drug entry and alteration in the target ribosomal protein are the other mechanisms involved in aminoglycoside resistance. Resistance to colistin is thought to be mediated with modifications of the lipopolysaccharides of the bacterial cell membrane that interfere with the agent’s ability to bind bacterial targets while decreased susceptibility to tigecycline has been associated with the overexpression of the AdeABC multidrug efflux pump, which confers resistance to various classes of antibiotics [22,23].

### 2.4. Stenotrophomonas maltophilia

*S. maltophilia* is a Gram-negative obligate aerobe rod, an opportunistic pathogen which has becoming increasingly common, particularly among immunocompromised subjects or patients admitted to intensive care units (ICUs). According to the data of the INFORM Network, *S. maltophilia* was the sixth most common pathogen isolated from patients with pneumonia admitted to ICUs, among 75 US medical centers between 2015 and 2017 [24]. A recent retrospective cohort study based on the Premier Healthcare Database reported that *S. maltophilia* was the most frequent carbapenem-resistant Gram-negative pathogen isolated from blood culture between 2010 and 2015, accounting for 30.3% of all isolates [25]. Several case series reported a mortality rate as high as 69% in patients with bloodstream infections due to *S. maltophilia*; the highest mortality rates were demonstrated in patients with hematological malignancies, previous chemotherapy, hypoalbuminemia, pneumonia, septic shock, inadequate initial antimicrobial therapy and quinolone resistance [26].

The high mortality associated to life-threatening infections caused by this microorganism is mainly due to the limited treatment options. The natural resistance to most beta-lactams is due to the production of two chromosomically encoded beta-lactamases, the L1 enzyme, which is a metallo-beta-lactamase, and L2 enzyme, a clavulanic acid-sensitive cephalosporinase [26]. Resistance to other antibiotic classes is mainly mediated by the expression of several efflux pumps, including members of the ATP-binding cassette (ABC) family, conferring resistance to aminoglycosides, macrolides and polymyxins, proteins belonging to the major facilitator superfamily (*MfsA*), targeting aminoglycosides, macrolides, tetracyclines, fluoroquinolones and rifampicin, or to the RND family, contributing to the resistance to fluoroquinolones, trimethoprim-sulfamethoxazole (TRS), tetracyclines, macrolides and chloramphenicol [27]. Additional determinants of resistance that can be acquired by *S. maltophilia* include the *qnr* genes, conferring resistance to fluoroquinolones though target protection, acetyltransferase (*aac*) or phosphotransferase (*aph*) inactivating the aminoglycosides, as well as the *sul* and *dfrA* genes, contributing to the resistance to TRS [26].

## 3. Available Treatments for Carbapenem-Resistant Gram-Negative Bacteria: Old Drugs and Novel Options

### 3.1. Ceftolozane/Tazobactam

Ceftolozane/tazobactam (CTT) belongs to the first wave of novel beta-lactam/beta-lactamase inhibitor combinations aimed at countering Gram-negative resistant bacteria [28]. Relying on the results of the ASPECT-cIAI and ASPECT-cUTI trials, it was approved by the Food and Drug Administration (FDA) in 2014 and by the European Medicines Agency (EMA) in 2015 [29,30]. Initial indications were for the treatment of complicated intra-abdominal infections (cIAIs) and complicated urinary tract infections (cUTIs), including pyelonephritis, in adult patients at a dosage of 1.5 g every 8 h. In 2019, the indications for ceftolozane/tazobactam expanded to include nosocomial pneumonia, following the results of ASPECT-NP study, a non-inferiority phase 3 trial comparing the efficacy and safety of the drug against meropenem [31]. Notably, the drug was administered at a doubled dosage compared to what was previous approved (3 g every 8 h) [31].

As comprehensively synthetized by Yahav and colleagues, ceftolozane/tazobactam shows activity against ESBL-producing *Enterobacterales* and several strains of *P. aeruginosa* with difficult-to-treat resistance (DTR-PA), but not towards Ambler Class A, B or D carbapenemases, and it has very limited activity versus *Acinetobacter spp*. and *Stenotrophomonas maltophilia* [32]. Ceftolozane has enhanced affinity for the penicillin-binding proteins (PBPs) of the bacterium, allowing tazobactam to target other beta-lactamases; therefore, it is significantly less affected by the changes in the porin permeability or efflux pumps [32]. In numerous microbiological surveys across the world, almost the totality of *P*. *aeruginosa* strains were susceptible to ceftolozane/tazobactam, with rates above 80% for MDR strains [33,34,35].

The most recent systematic review on the real-world use of ceftolozane/tazobactam included 83 studies (up to June 2020) comprising 3701 patients: 90.7% of them had a *P. aeruginosa* infection, with good clinical and microbiological responses in spite of the high heterogeneity regarding types of infections and patients, the level of resistance expressed by the pathogen and the different schedules used [36].

As better explained in the dedicated paragraph, ceftolozane/tazobactam has become one of the reference drugs for *P. aeruginosa* infections in the case of resistance to all first-line options (quinolones, beta-lactams such as cefepime and piperacillin/tazobactam) plus carbapenems. Although related evidence from a randomized clinical trial is lacking, there are interesting data from observational studies informing this recommendation. In a retrospective, multicenter, observational cohort study comparing patients treated with ceftolozane–tazobactam with those treated with either polymyxin or aminoglycoside-based regimens for infections due to drug-resistant *P. aeruginosa*, subjects receiving ceftolozane/tazobactam shower higher clinical cure (adjusted odds ratio [aOR]: 2.63; 95% CI, 1.31–5.30) and lower acute kidney injury (aOR: 0.08; 95% CI: 0.03–0.22), after adjusting for differences in baseline characteristics between groups [37]. The incidence of nephrotoxicity was 6% in the ceftolozane/tazobactam arm versus 34% in the comparator group. Notably, in the ceftolozane/tazobactam arm, combination therapy was used only in 15% of cases, whereas in the comparator group, combination was more common (72%) [37].

The emergence of resistance to ceftolozane/tazobactam is becoming a not-negligible issue, with potential cross-resistance involving other novel beta-lactams such as ceftazidime/avibactam. Resistance is most commonly owing to amino acid substitutions, insertions or deletions in *Pseudomonas*-derived cephalosporinase (PDC), the chromosomally encoded class C β-lactamase of *P. aeruginosa*, usually named as “the pseudomonal Ampicillinase C (AmpC)” [38]. In a recent study, it is suggested that development of resistance in patients treated with ceftolozane/tazabactam for carbapenem-resistant *P. aeruginosa* may be driven by inadequate source control and by intermittent instead of extended (3 h) infusion [39].

Eventually, its place in therapy may also include infections by Extended Spectrum Beta-lactamase (ESBL)-producing Enterobacterales. In a multicenter Italian cohort of 153 subjects treated with ceftolozane/tazobactam (48.3% in intensive care units), used as empiric therapy in 46 (30%) patients and as monotherapy in 127 (83%) patients, clinical success was observed in 78.3% of the cases, whereas 30-day mortality was 9.8% [40]. In multivariable analysis, receiving ceftolozane/tazobactam as empiric therapy (OR, 0.12; 95% CI, 0.01–0.34) was the sole factor associated with clinical success, along with an adequate source control of the infection (OR, 0.42; 95% CI, 0.14–0.55) [40]. Thus, ceftolozane/tazobactam may come in useful for infections due to ESBL-producing strains against the backdrop of a carbapenem-sparing strategy [41].

### 3.2. Ceftazidime/Avibactam

Ceftazidime/Avibactam (CTV) is a ß-lactam/ß-lactamase inhibitor (BL/BLI) combination approved by the Food and Drug Administration (FDA) and the European Medicines Agency (EMA) for the treatment of complicated intra-abdominal infections (cIAI), complicated urinary tract infections (cUTI), hospital-acquired pneumonia (HAP) and ventilator-associated pneumonia (VAP) [42,43]. Ceftazidime (CTZ) is a third-generation cephalosporin with a broad spectrum of activity against Gram-negative bacilli, including *Pseudomonas aeruginosa* [44]. Avibactam (AVI) is a non-ß-lactam-BLI which has a different mechanism of action compared with other BLIs [45]. AVI indeed lacks a beta-lactam ring and exploits a reactive urea to inhibit class A ESBLs and carbapenemases (e.g., *K. pneumoniae* Carbapenemase, KPC) as well as some class C and class D betalactamases (e.g., AmpC, OXA) [46]. In fact, AVI was shown in in vitro study to potently inhibit all tested class A BLs (including TEM-1, CTX-M-15 and KPC-2) and to restore the susceptibility to cephalosporins and aztreonam against bacteria producing CTX-M, KPC, amp-C and OXA-48 beta-lactamases [46,47,48,49]. On the contrary, AVI did not show such an effect against MBL-producing bacteria [50]. It should also be stressed that neither AVI nor its combination with CTZ showed efficacy against *Acinetobacter baumannii* [51]. Moreover, Enterobacterales can develop resistance to CTV through mutations of both the KPC and AmpC genes, as well as through porin loss, while *P. aeruginosa* can develop resistance against CTV through mutations in porin-encoding genes and overexpression of efflux pumps [52,53,54,55]. According to the INFORM global surveillance 2012–2015 study, >95% of tested Enterobacterales showed in vitro susceptibility to CTV (except for MBL-producing Enterobacterales), with a subset of isolates non-susceptible to ceftazidime or meropenem. It was effective against MBL-negative Enterobacterales with all combinations of class A, C and D BL, including KPC and OXA-48 [56]. The clinical indications for the use of CTV came from several phase 3 trials conducted among patients with complicated cIAI (RECLAIM, REPRISE), cUTI (REPRISE, RECAPTURE) and HAP/VAP (REPROVE). In the RECLAIM study, CTV plus metronidazole showed non-inferiority to meropenem in the treatment of cIAI with a difference in clinical cure rate at time of cure (TOC) of −2.4 (95%CI: −6.90 to 2.10) in the modified intention-to-treat (MITT) population, and of −3.5 (95%CI: −8.64 to 1.58) in the microbiologically MITT (mMITT) population [57]. In the REPRISE study, adult inpatients with cIAI or cUTI with evidence of infections caused by ceftazidime-resistant bacteria were treated with CTV or with best available therapy (BAT) for 5–21 days; CTV was associated with metronidazole in patients with cIAI [58]. Globally, CTV showed non-inferiority to BAT in patients with either cIAI or cUTI (clinical cure at TOC: 91% [95%CI: 85.6–94.7] vs. 91% [95%CI: 85.9–95.0%]). CTV was also compared with doripenem for treatment of cUTI in the RECAPTURE trial, in which the non-inferiority of CTV was also demonstrated [59]. Finally, in the REPROVE study, CTV was effective in the treatment of adult inpatients with HAP (including VAP), showing non-inferiority to meropenem, with differences in clinical cure at TOC of −4.2 (95%CI: −10.79 to 2.46) in the clinically MITT population, and of −0.7 (95%CI: −7.86 to 6.39) in the clinically evaluable population [60]. In all these studies, CTV was administered at a dosage of 2000 mg of CTZ plus 500 mg of AVI intravenously every 8 h. In an Italian retrospective observational multicenter study, CTV was confirmed to be effective in infections caused by carbapenemases-producing *K. pneumoniae* [61]. The mortality rate of the 577 inpatients included in the analysis was of 26.1% among patients receiving CTV alone and of 25.0% (*p* = 0.79) in patients treated with a CTV-based combination treatment. Mortality was associated with the presence of septic shock and neutropenia and was lower among patients treated with prolonged infusion of CTV. Similar results were shown in a systematic meta-analysis conducted among studies evaluating efficacy of CTV, alone or in combination, in the treatment of CRE and carbapenem-resistant *P. aeruginosa* [62]. Mortality rates of 30.9% and of 38.1% were indeed reported for CTV monotherapy and combination treatment, respectively (Relative Risk (RR): 1.18; 95CI: 0.88–1.58, *p* = 0.259). Moreover, no differences in rates of microbiological cure were reported in the two groups (63.4% vs. 64.9%; RR: 1.05; 95CI: 0.85–1.28, *p* = 0.705).

### 3.3. Meropenem/Vaborbactam

Meropenem/Vaborbactam (MEV) is the combination of the widely known carbapenem meropenem with the new-generation beta-lactamase inhibitor (BLI)Vaborbactam. Meropenem and other carbapenems have been extensively used for the treatment of Enterobacterales resistant to third and fourth generation cephalosporins in the past years. However, due to the alarming increase in the rate of carbapenem-resistant Enterobacterales, the development of new drugs with activity against these strains became necessary. MEV was initially approved by the FDA in 2017 for the treatment of cUTI, including pyelonephritis at a dosage of 2 g (1 g of Meropenem plus 1 g of Vaborbactam) i.v. every 8 h [63]. In 2018, MEV was also approved by the EMA for treatment of cUTI, cIAI and HAP (including VAP) at the same dosage [64].

Some authors found that MER-based combinations are almost effective in treating CRE when MER is given as high-dose continuous infusions, but this therapeutic conduct is controversial [65]. VAB is a non-ß-lactam serine BLI based on a cyclic boronic acid pharmacophore with potent activity especially against KPC [66]. These characteristics make VAB different from other BLI that also inhibit KPC, such as avibactam and relebactam. The cyclic boronic acid possesses affinity for the serine-based active sites of BL across the formation of a covalent complex which prevents the hydrolysis by serine carbapenemases [66]. VAB showed potent activity against class A and class C BLI, especially against KPC, but it has no activity against other Amber’s Class BLI such as class B and class D carbapenemases. In fact, in vitro studies showed very low MICs of the MEV combination for all tested Enterobacterales, which were known to be KPC-positive, OXA-48-negative and MBL-negative [67]. On the contrary, MEV showed little efficacy against MBL-producing CRE, DTR *P. aeruginosa*, *S. maltophilia* and *A. baumannii* in in vitro study [68,69,70]. The clinical efficacy of MER/VAB was evaluated in two phase 3 RCTs: TANGO I and TANGO II. In the TANGO I study, 550 patients with cUTI or acute pyelonephritis were randomized and allocated in a 1:1 ratio to receive MEV 2 g/2 g i.v. every 8 h or Piperacillin/Tazobactam (PIT) 4 g/0.5 g i.v. every 8 h. [71]. MEV met the non-inferiority endpoint with an efficacy difference in the mMITT population of 4.5% (95CI: 0.7–9.1%, *p* < 0.001). Microbiological eradication in the mMITT population occurred in 66.7% of patients in the MEV group and in 57.7% of patients in the PIT group (difference, 9.0% [95CI: −0.9% to 18.7%]). Combining the different endpoints, the overall success in the MEV group was 74.5% vs. 70.3% in the PIT group (difference, 4.1% [95CI: −4.9% to 9.1%]). In the TANGO II study, patients with confirmed or suspected CRE infections were enrolled [72]. Eligible patients were those with cUTI/acute pyelonephritis, HAP/VAP, cIAI or bacteremia. Seventy-seven patients were randomized 2:1 to MER/VAB or BAT. MEV was given i.v. at a dosage of 2 g/2 g every 8 h for 7–14 days. Efficacy in the microbiological-CRE-modified ITT (mCRE-MITT) population at TOC was higher for MEV than for BAT (59.4% vs. 26.7%; difference, 32.7%; 95CI: 4.6–60.8%; *p* = 0.02). Moreover, all-cause mortality at day 28 was lower in the MER/VAB group than in the BAT group (15.6% vs. 33.3%; difference −17–7%; 95CI: 44.7–9.3%; *p* = 0.20). Results from a real-world retrospective multicenter study conducted in the United States showed a 30-day mortality of 18.3% among 126 patients with Gram-negative bacterial infections, including CRE [73]. Mortality at day 30 was similar in patients with confirmed CRE infections (*n* = 99, 19.2%). The rate of patients who showed a worsening of clinical condition or a failure to improve while on treatment with MEV was 23.8%, while it was 25.8% in patients with confirmed CRE infections. The most common sources of infections were respiratory tract (38.1%), cIAI (19.0%) and urinary infections (13.1%). In a multicenter retrospective study of adults with CRE infections, no differences in clinical success were reported in patients treated with MEV (69.2%) compared with patients treated with CTV (61.1%, *p* = 0.49), although patients in the MEV group (*n* = 13) were fewer than patients in the CTV group (*n* = 90) [74]. The mortality rate at day 30 (11.5% vs. 18.1%, *p* = 0.57) and at day 90 (26.9% vs. 28.6%, *p*= 0.48) was similar between the two groups.

### 3.4. Imipenem/Cilastatin/Relebactam

Imipenem/cilastatin/relebactam (IMR) links together the carbapenem imipenem, the renal dehydropeptidase-I inhibitor cilastatin (having no antimicrobial activity but reducing the renal metabolism of imipenem) and the novel beta-lactamase inhibitor relebactam [75]. The latter one can protect imipenem from degradation by Ambler class A and class C beta-lactamases and from *Pseudomonas*-derived cephalosporinase (PDC); however, relebactam is not active against class B metallo-beta-lactamases or class D oxacillinases [70]. At any rate, the addition of relebactam does not enhance the activity of imipenem against *A. baumannii* and *Stenotrophomonas maltophilia* [76]. Imipenem/cilastatin/relebactam is approved in the United States, as well as in the European Union, in adults for the treatment of nosocomial pneumonia, cUTIs, cIAIs and other infections by antibiotic-resistant Gram-negative pathogens (essentially carbapenem-resistant Enterobacterales and *Pseudomonas*) in the case of limited or no alternative treatment options [75].

The efficacy and safety of the drug have been tested in two Phase 3 RCTs, RESTORE-IMI 1 and RESTORE-IMI 2, while limited real-life data are available in literature [77,78,79].

RESTORE-IMI 1 compared imipenem/cilastatin/relebactam versus a combination of imipenem and colistin for different types of infections brought on by imipenem non-susceptible pathogens such as nosocomial pneumonia (including the ventilator-associated forms), cUTI and cIAI [77]. Overall, 47 patients were included in the study: 31 in the arm of the investigational drug and 16 in the other one; the mMITT population was smaller, 21 versus 10 subjects, and *P. aeruginosa* accounted for 77% of cases, the rest being represented by Enterobacterales. The primary endpoint was a favorable clinical response in the mMITT population: most mMITT patients fulfilled the outcome, 71% in the investigational group and 70% in the comparator arm (adjusted difference, –7.3%; 90% CI, –27.5–21.4%) [77]. Interestingly, 28-day all-cause mortality was 20% lower with imipenem/cilastatin/relebactam (9.5% versus 30%, adjusted difference, –17.3%; 90% CI, –46.4–6.7%) [77]. Of the 47 patients belonging to the overall population, 45 had enough data to assess nephrotoxicity: no patients undergoing imipenem/cilastatin/relebactam experienced kidney failure according to Risk, Injury, Failure, Loss, and End-stage Kidney Disease (RIFLE) criteria, as opposed to the group undergoing imipenem plus colistin (25%) [80].

RESTORE IMI-2 evaluated the non-inferiority of imipenem/cilastatin/relebactam in comparison with piperacillin/tazobactam for the treatment of hospital-acquired/ventilator-associated bacterial pneumonia (HABP/VABP) in hospitalized adults [78]. Of 537 randomized subjects, the MITT population consisted of 264 imipenem/cilastatin/relebactam and 267 piperacillin/tazobactam patients. The most common causative agents were *K. pneumoniae* (25.6%) and *P. aeruginosa* (18.9%). Imipenem/cilastatin/relebactam was non-inferior to piperacillin/tazobactam in the MITT population as to the primary outcome of 28-day all-cause mortality (adjusted treatment difference 5.3%; 95% CI, –11.9 to 1.2%) [78]. Of note, 28-day mortality was lower in two predefined subgroups: mechanically ventilated patients (adjusted treatment difference –11.2%; 95% CI, –21.6 to –0.5%) and those with an APACHE II score equal/greater than 15 (adjusted treatment difference –15.4%; 95% CI, –26.2 to –4.4%) [78].

Lastly, the emergence of resistance towards this new drug has already been described: currently, changes in permeability and efflux are deemed the primary drivers of non-susceptibility [81].

### 3.5. Cefiderocol

Cefiderocol is a novel siderophore-cephalosporin antibiotic that received FDA approval for the treatment of urinary tract infections and nosocomial pneumonia, both hospital-acquired pneumonia and ventilator-associated bacterial pneumonia. It is active in vitro on aerobic fermentative and non-fermentative multidrug-resistant (MDR) Gram-negative rods.

Cefiderocol is a synthetic conjugate composed of a cephalosporin moiety and a catechol-type siderophore, which binds to iron and facilitates bacterial cell entry using active iron transporters, utilizing a “Trojan horse” approach [82]. Once inside the periplasmic space, it dissociates from iron and the cephalosporin moiety binds primarily to penicillin-binding protein 3 to inhibit bacterial cell wall synthesis [83]. The ability to be actively transported within the cell gives cefiderocol the ability to overcome resistance mechanisms due to a reduction in the permeability of the bacterial membrane resulting from reduced expression or mutation of porin channels and/or upregulation of the efflux pumps and inactivation by carbapenemases [82,84,85]. More specifically, in carbapenem-resistant Enterobacterales, cefiderocol has been shown to have activity against extended spectrum beta-lactamases (ESBLs), such as CTX-type, SHV-type and TEM-type, as well as all Ambler classes of beta-lactamases, including class A (KPC), class B (NDM, IMP and VIM), class C (AmpC) and class D (OXA, OXA-24, OXA-48, and OXA-48-like) [86]. Its broad spectrum of action and its extended activity on all carbapenemases makes cefiderocol a drug that could be reserved for patients with limited or no alternative therapeutic options in order to prevent widespread resistance or as empirical treatment in high-resistance settings.

The global surveillance studies, SIDERO-WT and SIDERO-CR, demonstrated a very high rate of susceptibility to cefiderocol compared with other β-lactam/cephalosporin antibiotics [67,87]. Analyzing data from the SIDERO surveillance studies [88], 236 strains not susceptible to carbapenems (MICs > 8 mg/L) showed susceptibility rates as high as 94.9% for cefiderocol [89]. Susceptibility rates over 90% are also reported for OXA-23- and OXA-24-like producers. However, in vitro studies showed the MICs for New Delhi metallo-β-lactamase (NDM) positive isolates to be significantly higher than those harboring other β-lactamase genes, with a susceptibility rate of 83.4% for cefiderocol [90]; the expression of NDM appears to facilitate the emergence of cefiderocol resistance by mutations in siderophore receptors [91,92].

As with other cephalosporins, the activity of cefiderocol is best described by time-dependent killing, which is enhanced when cefiderocol is administered as an extended 3 h infusion compared to a 1 h infusion [93].

### 3.6. Colistin

Colistin is an old antimicrobial agent available since the 1950s that belongs to the polymyxin antibiotic class which consists of five polymyxins, A, B, C, D and E, where colistin is the polymyxin E. It shows activity against a variety of Gram-negative rods, such as *A. baumannii*, *P. aeruginosa*, *E. coli* and *K. pneumoniae* MDR, while it is not active, due to intrinsic resistance, against *Serratia* spp., *Proteus* spp., *Morganella* spp. and *Providencia* spp. Their clinical use has recently resurfaced as salvage therapy for difficult-to-treat MDR and extensively drug-resistant (XDR) Gram-negative infections, due to the emergence of superbugs, which are resistant against all other available antibiotics [94]

Colistin is a bactericidal agent mainly active against GNB due to the presence of lipopolysaccharide (LPS) in the GNB cell wall [95]. Indeed, polymyxins have a unique mechanism of action involving disruption of the outer membrane integrity of Gram-negative bacteria that, in addition to providing rapid bactericidal activity, may enhance the activity of other antibiotic classes [96].

Colistin sulfate is available for oral or topical use or use with an inhalator. In the case of pneumonia, the aerosolized route of administration is favorable, as it presumably delivers a high concentration of drug directly to the infection site.

Colistin, for parenteral use, is administered as an inactive prodrug, colistimethate (also known as colistin methanesulfonate [CMS]) which makes it the preferred polymyxin for the treatment of lower urinary tract infections, given renal clearance of the prodrug CMS that then converts to the active moiety colistin in the urinary tract [97].

Unfortunately, its use is limited because it is characterized for a narrow therapeutic window due to its nephrotoxicity that occurs frequently with conventional doses and neurotoxicity.

The main mechanism of resistance is represented by the replacement of the phosphate groups of lipid A with cationic moieties, mediated by the acquisition of *mcr* genes. The SENTRY study reported a prevalence of colistin resistance of 1.7% among over 21,000 clinical isolates of *E. coli* and *K. pneumoniae* collected worldwide in 2014 and 2015 [98]. A recently published meta-analysis including 218 observational studies estimated a 7.4% prevalence of resistance among *E. coli* strains isolated from healthy individuals and a 4.2% prevalence among clinical isolates [99].

### 3.7. Fosfomycin

Fosfomycin is an old antibiotic which has been progressively repurposed in recent years as a combination partner in the therapeutic management of both Gram-positive and Gram-negative MDR and XDR bacteria.

Fosfomycin is a bactericidal antibiotic agent that plays an important role in urinary tract infections by reducing the adherence of bacteria to urinary epithelial cells [100].

Aside from its use for urinary tract infections, fosfomycin was predominantly used for sepsis/bacteraemia, respiratory tract, central nervous system and bone and joint infections. Indeed, its ability to penetrate into biofilms and its action not only have been able to reduce or eradicate clinically significant bacteria from biofilms, but also resulted in modifications of the biofilm structure [100,101].

Fosfomycin is active against many problematic Gram-positive pathogens such as *Enterococcus* spp. (including *Enterococcus faecalis* and *E. faecium* irrespective of vancomycin resistance), *Staphylococcus aureus* (irrespective of methicillin resistance) and *S. epidermidis* [102,103]. Fosfomycin also exhibits considerable activity against *Enterobacteriaceae* (e.g., *Salmonella* spp., *Shigella* spp., *E. coli*, *Klebsiella* spp., *Enterobacter* spp., *Serratia* spp., *Citrobacter* spp. and *Proteus mirabilis*) [100,104] while showing no activity, due to intrinsic resistance, against non-fermenting Gram-negative bacteria (e.g., *Pseudomonas* spp., *Acinetobacter* spp., *Stenotrophomonas maltophilia* and *Burkholderia cepacia*) [105,106]. It also shows only very limited activity against anaerobic species [107]. However, in some studies, Fosfomycin has been shown to reduce MIC to other antibiotics, thus restoring their susceptibility for non-fermenting bacteria [108].

It is not fully elucidated whether bacterial killing with fosfomycin is time- or concentration-dependent. According to in vitro data, it displays time-dependent killing against *P. aeruginosa* and *S. aureus*, while concentration-dependent activity was demonstrated against *E. coli* [109,110].

### 3.8. Aminoglycosides

Aminoglycosides were among the first antibiotics to be introduced in clinical practice, but their use declined due to safety concerns and the availability of many other alternative options. With the current rise in antimicrobial resistance, such an “old” antibiotic class is receiving renewed clinical interest. [111].

Moreover, a novel agent in this class has been quite recently approved by FDA for cUTI including pyelonephritis: plazomicin, that has demonstrated potent in vitro activity against ESBL-producing and carbapenem-resistant Enterobacterales [112]. Plazomicin is protected from nearly all clinically relevant aminoglycoside-modifying enzymes (AMEs), allowing it to overcome the limitations in the management of infections by antibiotic-resistant Enterobacterales typical of older aminoglycosides [112]. On the other hand, plazomicin does not show improved activity over traditional aminoglycosides against other superbugs among the Gram-negative bacteria, such as *P. aeruginosa* and *A. baumannii.*

Two clinical studies have been conducted in patients with cUTI comparing intravenously plazomicin 15 mg/kg once-daily with meropenem 1 g every 8 h and levofloxacin 750 mg once-daily, the first being a Phase 3 and the other a Phase 2 trial [113,114]. Both studies showed non-inferiority of plazomicin. A third study specifically focused on invasive infections by carbapenem-resistant Enterobacterales, poorly represented in the afore-mentioned trials, was stopped prematurely due to slow enrolment of patients: plazomicin-based therapy was compared with colistin-based regimens [115,116]. Of note, the pooled analysis of these trial studies showed that the rates of plazomicin and comparator-associated nephrotoxicity were 4.8% and 4.1%, respectively; therefore, plazomicin was not associated with a higher risk of nephrotoxicity than that of the comparator [117].

At any rate, plazomicin is not available in most European markets after the withdrawal of its application by the related company to the European Medicines Agency (EMA), advancing pharmacoeconomic considerations [118].

Therefore, indications from the Infectious Diseases Society of America (IDSA) guidelines back the use of single-dose aminoglycosides for uncomplicated cystitis, both for carbapenem-resistant Enterobacterales and for difficult-to-treat resistance (DTR) *P. aeruginosa*, if susceptibility is demonstrated [12]. European guidelines recommend, although with low certainty of evidence, aminoglycosides as monotherapy for non-severe infections by carbapenem-resistant Enterobacterales in the case of a favorable source such as the urinary tract; otherwise, on case-by-case basis and according to the susceptibility testing results, aminoglycosides may be used in the framework of a combination therapy, and the same reasoning applies to DTR-*P. aeruginosa* [13]. In conclusion, aminoglycosides in these scenarios may be taken into account for simplified treatment or when other drugs are not allowed.

### 3.9. Antibiotics in the Pipeline for the Treatment of MDR-GNB Infections

In this section we will provide a brief overview on antibiotics that have not reached or completed phase 3 of development.

Durlobactam is a diazabicyclooctanone (DBO) b-lactamase inhibitor that has been studied in combination with sulbactam and has intrinsic activity against *A. baumannii* (through affinity to PBP1 and PBP3) in an ongoing phase 3 trial. This beta-lactam/beta-lactamase inhibitor (BL/BLI) combination, not yet FDA-approved, demonstrates activity against Ambler class A, C and D β-lactamases with potential utility for carbapenem-resistant *A. baumannii* (CRAB). The phase 3 trial designed to study pneumonia and bacteremia caused by *A. baumannii–calcoaceticus* complex infections compares sulbactam-durlobactam plus imipenem versus colistin plus imipenem [119].

The spectrum of activity of cefepime/tazobactam includes Enterobacterales that are AmpC-, ESBL-, K1-, or OXA-48-β-lactamase-producing, while KPC- and NDM-producing Enterobacterales are mostly resistant [32]. An interventional RCT comparing cefepime–tazobactam versus meropenem for cUTI is ongoing, while clinical experience is reported only from India in a retrospective study in which clinical improvement was documented in 142 patients (92.2%) among 154 patients [120,121].

Cefepime–taniborbactam inhibits class A, C, D and even class B β-lactamases, including VIM, NDM, SPM-1 and GIM-1 (but not IMP) [32]. The combination cefepime–taniborbactam has been demonstrated to provide potent activity against strains with an elevated MIC to ceftazidime–avibactam and it was recently demonstrated to have potent in vitro activity against Enterobacterales and *P. aeruginosa*. A phase 3, randomized, double-blind noninferiority study is currently recruiting patients to evaluate cefepime–taniborbactam versus meropenem for the treatment of cUTI in adults [120].

Cefepime–enmetazobactam is a combination that was shown to be as effective as carbapenems against ESBLs in vitro. In addition, the combination is active against class A, C and D β-lactamases, while it does not enhance the potency of cefepime against *P. aeruginosa*. An ongoing phase 3, randomized, controlled, double-blind noninferiority trial is currently recruiting adult patients with cUTI for treatment with cefepime versus piperacillin/tazobactam [120,122].

Finally, aztreonam/avibactam is able to inhibit cell wall synthesis in metallo-beta-lactamase (MBL)-producing strains and showed potent in vitro activity against Enterobacterales with class B, A, C and some D beta-lactamases. Neither aztreonam alone nor the combination of aztreonam–avibactam has in vitro activity against A. *baumannii* [49]. A phase 3 randomized controlled trial comparing aztreonam/avibactam with or without metronidazole versus meropenem with or without colistin for the treatment of HABP/VABP and cIAI is ongoing [32,123].

### 3.10. Treatment Strategies for Carbapenem-Resistant Gram-Negative Bacteria

#### Carbapenem-Resistant Enterobacterales (CRE)

Before the availability of the newest beta-lactam antibiotics such as CTV and MEV, treatment of infections caused by CRE consisted of salvage combination therapies based on colistin, carbapenem-containing combinations or a double carbapenem combination [124]. According to the INFORM global surveillance study on in vitro susceptibility to CTV, most of the meropenem non-susceptible Enterobacterales were found to be susceptible to CTV (83.5%; MIC50: 1 µg/mL; MIC90: >128 µg/mL) [125]. The susceptibility rate to CTV was higher among MBL-negative CRE (97.7%; MIC50: 1 µg/mL; MIC90: 4 µg/mL), and OXA-48-like-positive MBL-negative CRE (98.5%; MIC50: 0.5 µg/mL; MIC90: 2 µg/mL). Conversely, the susceptibility rate to CTV of MBL-positive CRE was 3.4% (MIC50: >128 µg/mL; MIC90: >128 µg/mL).

MEV was specifically developed to target KPC-producing CRE [126]. A European survey among clinical isolates of inpatients with pneumonia showed that most isolated Enterobacterales were susceptible to MEV (98%; MIC50: 0.03 µg/mL; MIC90: 0.06 µg/mL) [68]. However, susceptibility to MER/VAB dropped to 63.0% (MIC50: 4 µg/mL; MIC90: >32 µg/mL) due to the presence of OXA-48- and MBL-producing strains of Enterobacterales. Susceptibility to MEV was indeed 99.1% (MIC50: 0.12 µg/mL; MIC90: 1 µg/mL) among KPC-producing CRE. Another in vitro study showed that most tested OXA-48-positive and MBL-positive Enterobacterales had MEV MICs of ≥16 µg/mL, thus confirming the inactivity of this combination against CRE producing Amber’s Class B and D carbapenemases [127]. IMR possess an activity spectrum against CRE which is similar to that of MEV. It is indeed active against class A carbapenemases, but it showed no activity against MBL and class D OXA-48 enzymes [128]. The SMART European surveillance report, conducted among clinical isolates of Enterobacterales from intra-abdominal and urinary tract infections, showed an overall in vitro susceptibility to IMI/REL of 98.4% [129]. The susceptibility rate dropped to 42.4% when only imipenem-non-susceptible strains were considered, but it was 98.6% among KPC-producing Enterobacterales. In most IMR-non-susceptible Enterobacterales the presence of MBLs (53.9%) or OXA-48-like enzymes (42.4%) were detected. In a subsequent report of the SMART study, which was also conducted among respiratory samples, isolated Enterobacterales showed a susceptibility rate of 95.2% (MIC50: 0.25 µg/mL; MIC90: 1 µg/mL) to IMR, while the susceptibility rate among IMR-non-susceptible Enterobacterales was 66.3% (MIC50: 1 µg/mL; MIC90: >32 µg/mL) [130].

From what has been discussed thus far, with the available paraphernalia of BL/BLI, it may be challenging to deal with infections due to MBL-producing Enterobacterales. A treatment option for MBL-producing CRE may be represented by combinations of aztreonam with new BL/BLI. Aztreonam is indeed effective against class B and D carbapenemases, but it is not active against ESBLs and class A carbapenemases, which are often concurrently carried by MBL-producing CRE [131,132]. The most studied combination is Aztreonam plus CTV, which showed in vitro activity against different strains of CRE, including NDM-producing *E. coli*, VIM-producing *C. freundii* and OXA-producing *A. baumannii* [133]. Furthermore, a study conducted among 40 MBL-producing *K. pneumoniae* isolates showed the presence of in vitro synergistic activity of Aztreonam combined with CTV (97.5%), MEV (72.5%) and also IMR (97.5%) [134].

Finally, the novel siderophore cephalosporine Cefiderocol represents a promising treatment option for MBL-producing Enterobacterales. In fact, an in vitro study conducted among MDR Gram-negative isolates showed that Cefiderocol inhibited 78.7% and 92.1% of MDR Enterobacterlaes isolates at MICs of 2 µg/mL (EUCAST breakpoint) and 4 µg/mL (CLSI breakpoint), respectively [135]. In particular, cefiderocol inhibited most isolates of Enterobacterales producing IMP (93.3% at MIC of 2 µg/mL; 100% at MIC of 4 µg/mL), VIM (80.9% at MIC of 2 µg/mL; 95.7% at MIC of 4 µg/mL) and OXA-48-like beta-lactamases (92.9% at MIC of 2 µg/mL; 98.2 at MIC of 4 µg/mL) and those expressing AmpC + porin loss (100% at either MIC 2 µg/mL and 4 µg/mL). The susceptibility rate to cefiderocol was lower among Enterobacterales harboring the NDM MBL (41% at MIC of 2 µg/mL; 72.1% at MIC of 4 µg/mL) and ESBL + porin loss (61.5% at MIC of 2 µg/mL; 88.5% at MIC of 4 µg/mL). The reduced susceptibility to Cefiderocol among NDM-producing CRE was confirmed in the SIDERO-WT-2014 global surveillance program [136].

### 3.11. Pseudomonas aeruginosa with Difficult-to-Treat Resistance (DTR-PA)

All the current guidelines [12,13,14] have tried to answer two fundamental questions: (i) the place in therapy of novel anti-pseudomonal agents, such as ceftolozane/tazobactam, ceftazidime/avibactam and imipenem/cilastatin/relebactam, cefiderocol; and (ii) the potential role of targeted combination therapy. The conclusions among the afore-mentioned documents are not uniform but the premises are the same: there is a lack of head-to-head trials comparing the novel available anti-pseudomonal agents against each other, and the evidence is substantially rooted in pre-clinical data, observational studies and randomized controlled trials (RCTs) of limited sample size [12,13,14].

For instance, ceftolozane/tazobactam is acknowledged as one of the most powerful new-generation anti-pseudomonal antibiotics; nevertheless, the pivotal RCTs allowing its introduction in clinical practice were not aimed at including carbapenem-resistant strains; in the ASPECT-NP study on nosocomial pneumonia, a small fraction of patients (15 out of 726 enrolled) were affected by XDR *P. aeruginosa*, and no information on CRPA was available [31]. Still, although the important limitations of polymyxins in terms of toxicity and suboptimal efficacy are widely known [137], no sufficiently powered RCT has been published demonstrating the clear-as-day inferiority of colistin-based therapy versus new anti-pseudomonal beta-lactams [13,14]. To this purpose, it is worth citing the pivotal RCTs regarding imipenem/cilastatin/relebactam and cefiderocol: the RESTORE-IMI trial and the CREDIBLE-CR study, respectively, both designed to assess the efficacy against carbapenem-resistant organisms of the novel drugs, the first having colistin (plus imipenem) as the comparator, the second a “best available therapy” based on colistin in two-thirds of cases concerning the carbapenem-resistant mITT population. [77,138]. In the former RCT, only 47 patients were recruited and invasive infections by DTR-PA accounted for 77% of the 31 cases representing the mITT: a favorable overall response (the primary endpoint) was observed in 81.3% versus 62.5% cases, but the adjusted difference was not statistically significant in this precise subset [77]. In the latter RCT, the all-cause mortality in the small DTR-PA subgroup (only 22 patients) was equal among the intervention and the comparator (18%) [138]. Therefore, these results do not bring forth rock-solid evidence of the apparent inferiority of colistin-based therapy for DTR-PA infections when compared with new-generation beta-lactams.

Nevertheless, beta-lactams have traditionally represented the mainstay of anti-pseudomonal treatment [139]; their convenient safety profile and their wide therapeutic window also make them preferable to other options in the setting of DTR-PA [12,13,14].

The second question involves the role of combination therapy. Historically, in contexts characterized by a high likelihood of *P. aeruginosa* as a causative agent, such as nosocomial pneumonia and febrile neutropenia, a dual anti-pseudomonal coverage was advocated as the empirical strategy, especially in the case of very serious infection or a high prevalence of resistant pathogens and considering that delaying appropriate therapy for about 48 h significantly raises the risk of 30-day mortality related to invasive *P. aeruginosa* infections [140,141,142]. Nonetheless, when it comes to definite treatment, monotherapy turns out not to be inferior compared with combination therapy. A recent meta-analysis on bloodstream infection or pneumonia by *P. aeruginosa* found no mortality difference among patients receiving targeted beta-lactam monotherapy and subjects undergoing combination therapy (relative risk 1.05, 95% confidence interval 0.86–1.28; *p* = 0.658) [143]. The same group of researchers had already demonstrated no difference in mortality regarding ceftazidime/avibactam for carbapenem-resistant GNB (including a limited number of DTR-PA cases) when used as monotherapy versus a combination regimen [62]. Recent observational data specifically on ceftolozane/tazobactam against drug-resistant *P. aeruginosa* highlighted how the receipt of the novel beta-lactam as monotherapy was independently associated with clinical cure on multivariable analysis and with a far better safety profile as opposed to polymyxin- or aminoglycoside-based therapies, often in combination regimens [37,144].

In Table 3 a summary of the current guidelines on DTR-PA infections is provided [12,13,14]. In the absence of head-to-head trials or robust observational data, it is difficult to prioritize one novel anti-pseudomonal beta-lactam over the others [12]. Pending a full susceptibility report, a useful aid may come from genotype testing: *P. aeruginosa* deploys a long list of resistance mechanisms, and in the framework of carbapenem resistance the role of carbapenemases, although not predominant as far as Enterobacterales are concerned, is becoming more relevant [145]. As synthetized by Karakonstantis and colleagues in a review, in settings where DTR-PA are not linked with carbapenemases production, ceftolozane/tazobactam and ceftazidime/avibactam may represent the first choice [146]. Imipinem/cilastatin/relebactam, like the two previous drugs, is not active against carbapenemases-producing strains, but may be an option in the case of AmpC mutations that confer resistance to ceftolozane/tazobactam and to ceftazidime/avibactam. Actually, the latter may be active in the case of GES-type beta-lactamases (class A carbapenemases), whereas the only beta-lactam currently authorized for clinical use active against infections by isolates producing metallo-beta-lactamases, the most frequent carbapenemases in DTR-PA, is cefiderocol [146].

### 3.12. Carbapenem-Resistant A. baumannii (CRAB)

Due to its difficult-to-treat resistance phenotype and the increased mortality rate compared with carbapenem-susceptible *A. baumannii* infections, the preferred treatment for CRAB infections is still controversial [147]. Treatment selection depends on the interpretation of in vitro efficacy, host factors, and pharmacokinetic–pharmacodynamic (PK/PD) data [16]. The emergence of resistance is limiting the use of the traditional agents used for CRAB infections such as polymyxins, sulbactam, tetracyclines and fosfomycin. The recent development of novel agents such as cefiderocol and eravacycline could expanded the armamentarium against CRAB infections.

Colistin represented, for several years, the therapeutic backbone for the treatment of severe *Acinetobacter* infections, although its use is limited by nephrotoxicity and neurotoxicity [148], suboptimal pharmacokinetics and poor pulmonary penetrability. The inhaled route of administration of colistin could be useful for CRAB infections involving the airways. Zheng et al., evaluating 183 patients with *A. baumannii* pneumonia treated with colistin, showed that inhaled colistin was the only independent predictor of 30-day survival, clinical response and microbiological eradication, unlike intravenous colistin, which appeared to be an independent predictor of clinical failure [149].

Several combination therapies with various antibacterial agents (such as carbapenems, sulbactam and fosfomycin) have been explored and compared with colistin monotherapy to treat drug-resistant *A. baumannii* infections.

An RCT [150] evaluated treatment with colistin in combination with meropenem and did not find better clinical improvement or lower mortality than colistin monotherapy in infections caused by carbapenem-resistant *A. baumannii.* Additionally, Huang et al., in a recent meta-analysis of 10 studies, provided evidence that colistin monotherapy was associated with similar rates of clinical improvement, 14-day mortality, hospital mortality, and nephrotoxicity to colistin plus meropenem combination therapy with low quality evidence that colistin plus meropenem combination therapy demonstrated a microbiological benefit [151].

In the meta-analysis by Kengkla et al., there was no statistically significant difference in clinical cure outcomes among colistin-based combinations with other antibiotic therapies in patients with MDR and XDR *A. baumannii* infections. However, colistin in combination with sulbactam was associated with a significantly higher microbiological cure rate than colistin monotherapy [152].

A retrospective study of patients diagnosed with CRAB pneumonia found that patients treated with colistin or ampicillin–sulbactam had similar clinical cure rates [153]. However, colistin was associated with higher rates of microbiologic failure, a reduction in renal function and increased 30-day mortality [153]. A recent systematic review found that high-dose sulbactam combined with additional anti-bacterial agents (including colistin) showed a certain level of efficacy for treating drug-resistant *A. baumannii* infections; the combinations were associated with significantly superior rates of microbiological evaluation when compared with colistin monotherapy alone or high-dose sulbactam combined with one additional antibacterial [154]. Finally, no combinations were associated with significant reductions in mortality from all causes, although nephrotoxicity that developed in response to drug combinations that included high doses of sulbactam was detected less frequently than in response to combinations that included colistin.

A study simulating in vitro triple therapy with high-dose minocycline, continuous-infusion sulbactam, and polymyxin B with a pharmacodynamic model found that this combination showed the most significant kill against CRAB, with no regrowth and minimal resistance development [155].

In a prospective, observational, multicenter study conducted from January 2017 to June 2020 including 180 hospitalized patients with severe pneumonia due to MDR-*A. baumannii*, a fosfomycin-containing regimen was independently associated with 30-day survival [156]. Additionally, Jung et al. [157] evaluated a fosfomycin-containing regimen for the treatment of pneumonia caused by drug-resistant *A. baumannii*, showing a more beneficial effect on all-cause mortality, with favorable effectiveness in clinical cure and microbiologic eradication [157]. Furthermore, in another study, a combination of colistin and fosfomycin had a significantly more favorable microbiological response and a trend toward more favorable clinical outcomes and lower mortality than those who received colistin alone [158]. Two studies evaluated the potential synergistic activity between fosfomycin and colistin against OXA-23-producing *A. baumannii*; they reported synergy against 50% of the strains in one study and 12.5% of strains in the other [159,160]. One of these studies reported synergy against 75% of strains when fosfomycin was combined with sulbactam [159].

Novel agents with in vitro activity against CRAB have been developed in recent years. The efficacy of cefiderocol was evaluated in two non-inferiority studies in patients with severe urinary tract infections (APEKS-cUTI) [161] or hospital-acquired pneumonia (APEKS-NP) [162] and in a randomized, open-label, phase 3 multicentre study (CREDIBLE-CR) [138].

APEKS-NP was a randomized, double-blind, phase 3, non-inferiority study conducted at 76 centers in Asia, Europe and the United States that found how cefiderocol was non-inferior to high-dose meropenem (2 g IV every 8 h, 3 h extended infusion) in critically ill patients with hospital-acquired pneumonia, with similar tolerability. A total of 19% of patients had infections with carbapenem-resistant pathogens (mainly *A. baumannii* or other *Acinetobacter* spp). Fourteen-day all-cause mortality, clinical cure, and microbiologic eradication were similar between treatment groups for participants infected with *A. baumannii* [162].

In the CREDIBLE-CR study [138], while cefiderocol had similar clinical and microbiological efficacy to BAT and clinical cure rates were higher with cefiderocol than with BAT, participants randomized to cefiderocol treatment had a significantly higher rate of all-cause mortality (34% vs.18%), mainly in the subgroup of patients with *Acinetobacter* infections. At the time of study enrollment, those infected with CRAB were older, with severe renal dysfunction and higher rates of intensive care unit admission and ongoing septic shock, so it cannot be excluded that discrepancies in the patients’ characteristics between arms could have influenced these results.

A recent observational retrospective study included patients with CRAB infections that were divided in two study groups according to the antibiotic treatment received: cefiderocol- and colistin-containing regimens. The authors found that thirty-day mortality was higher in patients receiving colistin compared to those who received cefiderocol-containing regimens (55.8% versus 34%, *p* = 0.018) [163]. Nephrotoxicity was more common in the colistin group. Microbiological failure occurred in 17.4% of patients receiving cefiderocol versus 6.8% of those receiving colistin (*p* = 0.079) [163]. Additionally, a retrospective multicentre observational study performed at four Italian hospitals on adult patients admitted to the ICU for severe COVID-19 and further diagnosed with CRAB infections found that cefiderocol was associated with a non-significant lower mortality risk (Hazard Ratio, HR 0.64, 95% CI 0.38–1.08, *p* = 0.10) [164].

Finally, Eravacycline is a tetracycline analogue that is FDA-approved for complicated intra-abdominal infections (cIAI). In vitro, eravacycline demonstrates lower MICs against CRAB than tigecycline and retains activity against isolates in the presence of OXA carbapenemases and colistin-resistant isolates [165,166]. Phase 3 cIAI trials demonstrated non-inferiority of eravacycline to both ertapenem and meropenem; however, *A. baumannii* infections only comprised 3% and 2% of the total study infecting pathogens, respectively [167,168].

### 3.13. Stenotrophomonas maltophilia

Although a real standard of care for the treatment of infections due to *S. maltophilia* has never been defined, cotrimoxazole has always been regarded as the drug of choice for this microorganism. This drug has retained a high level of activity against most strains, with rates of susceptibility ranging from 90% to 100% according to the largest surveillance studies [169]. However, the most recent reports from the SENTRY Surveillance Program have described a slight decrease in the susceptibility rate among 6467 clinical isolates of *S. maltophilia*, from 97.2% in 2001–2004 to 95.7% in 2013–2016 [170]. A recently published retrospective cohort study including a total of 284 patients with *S. maltophilia* infections reported a 36% rate of clinical failure and 15% 30-day mortality among the 217 subjects receiving TRS monotherapy [171].

Other antibiotics commonly used to treat *S. maltophilia* infections are fluoroquinolones. According to the data of the SENTRY Program, approximately 81% of isolates were susceptible to levofloxacin [170]. In a systematic review and meta-analysis conducted by Ko et al. including 663 patients with *S. maltophilia* infections treated with either a fluoroquinolone or trimethoprim-sulfamethoxazole, a slightly lower mortality rate was demonstrated in the group receiving fluoroquinolones (OR 0.62, 95% CI 0.39–0.99) [172]. This difference was not observed when comparing the single molecules (levofloxacin or ciprofloxacin vs. TRS). However, the lower genetic barrier and the higher risk of selecting resistant strains during treatment render the fluoroquinolones a less attractive option for severe infections [173].

A less commonly prescribed but certainly interesting treatment choice is represented by minocycline. Among a total of 1289 strains of *S. maltophilia* collected from 2014 to 2018 from 87 US medical centers, minocycline demonstrated a 99.5% susceptibility rate, retaining a high rate of activity (92.8%) even against TMP/SMX-resistant isolates [174]. Although limited clinical data are available in the literature, minocycline has reported promising results, with a 31% rate of treatment failure and 5% 30-day mortality among 39 patients treated in a recent retrospective study.

Considering its stability against both serine and metallo-beta-lactamases, cefiderocol has rapidly emerged as one of the most promising candidates for anti-Stenotrophomonas treatment. Several data confirmed the high in vitro activity of cefiderocol against this pathogen [175,176]. The SIDERO-WT study, including 127 clinical isolates of *S. maltophilia* collected in Italy from 2014 and 2018, reported a 100% susceptibility of cefiderocol against the tested strains [177]. Similarly, a retrospective study collecting 100 strains isolated from blood culture in Taiwan reported a MIC_90_ of 0.25 mg/L, with a range from ≤0.03 to 1 [176]. Unfortunately, clinical data on the efficacy of cefiderocol against *S. maltophilia* are limited. In the CREDIBLE-CR study [138] only five patients with infections due to this microorganism were included, and all of them had pneumonia and were assigned to the cefiderocol arm. At the end of the study, four out of five (80%) died, but no clear conclusion can be drawn from these data.

Aztreonam displays a unique stability against metallo-beta-lactamases, so the activity of aztreonam against L1 metallo-beta-lactamase, combined with the inhibition of the L2 enzyme by avibactam, can be used against *S. maltophilia*. Several reports have demonstrated that avibactam is able to restore the in vitro activity of aztreonam against *Stenotrophomonas* strains [178,179,180]. The aztreonam–avibactam combination was tested against 1839 *S. maltophilia* isolates collected worldwide, showing potent antimicrobial activity, including against TRS -resistant strains [181]. However, the clinical data are still limited to a few case reports, demonstrating the successful use of the aztreonam and ceftazidime/avibactam combination for bloodstream infections due to *S. maltophilia* [182,183].

### 3.14. Treatment Algorithm Description

**Figure 1:**
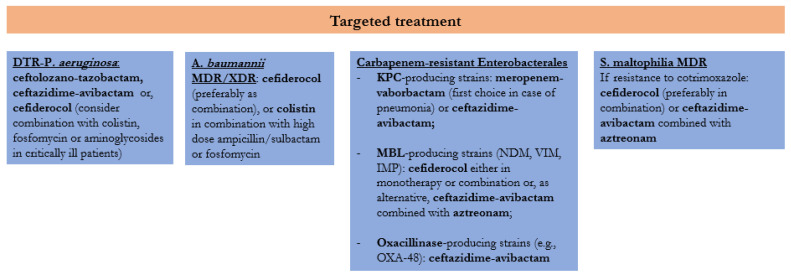
Treatment algorithm for infections due to carbapenem-resistant Gram-negative bacteria. **DTR**: Difficult-to-Treat Resistance; **MDR**: Multidrug Resistant; **XDR**: Extensively Drug Resistant; **KPC**: *Klebsiella Pneumoniae* Carbapenemase; **MBL**: Metallo-β-lactamase; **NDM**: New Dehli Metallo-β-lactamase; **VIM**: Verona Integron-encoded Metallo-β-lactamase; **IMP**: Imipenemase.

Our therapeutic algorithm for the targeted treatment of infections due to carbapenem-resistant Gram-negative bacteria is displayed in Figure 1. For DTR-*P. aeruginosa* we propose as first-line regimens ceftolozane–tazobactam or ceftazidime–avibactam, according to the susceptibility profile and the potential production of carbapenemases or other resistance mechanisms, and cefiderocol as alternative treatment. Moreover, we consider the possibility of adding a second active agent, among colistin, fosfomycin or an aminoglycoside, in critically ill patients. Several preclinical data have demonstrated a synergistic effect of combination based on a beta-lactam associated to fluoroquinolones, aminoglycosides, colistin, fosfomycin or rifampicin [184] against MDR *P. aeruginosa*. However, combination therapy has never been demonstrated to reduce mortality or improve microbiological outcomes in clinical studies [143,185,186,187].

For infections due to carbapenem-resistant A. *baumannii*, our algorithm suggests the use of cefiderocol, preferably as a combination, or colistin in combination with high-dose ampicillin–sulbactam or fosfomycin with or without meropenem. Despite a quite high mortality rate reported in the CREDIBLE-CR trial among patients with CRAB infections receiving cefiderocol [138], the drug was proved to be a good treatment option in this setting in a subsequent retrospective study, resulting in a lower mortality rate as compared to colistin, which was mostly used in monotherapy or in combination with tigecycline [163]. Although a colistin-based combination has demonstrated no clear benefit over colistin monotherapy against A. *baumannii* [188], the only prospective study showing a superiority of the combination strategy included ampicillin–sulbactam in the experimental arm [189].

Regarding the treatment of KPC-producing Enterobacterales, ceftazidime–avibactam and meropenem–vaborbactam are included in the algorithm, the latter being a preferred option in patients with pneumonia because of the higher concentrations achieved in the epithelial lining fluid [190]. For strains harboring metallo-beta-lactamase, the proposed options include cefiderocol, either in monotherapy or combination, or ceftazidime–avibactam associated with aztreonam, according to the indication of the most recent IDSA guidelines [191]. The latter option demonstrated a therapeutic advantage as compared to other active agents in a multicentre prospective study including 102 patients with bloodstream infections due to NDM- or VIM-producing Enterobacterales [192]. For strains producing class D carbapenemases ceftazidime–avibactam proved to be a valid option, while vaborbactam and relebactam display no activity against oxacillinases [193].

Finally, for the treatment of infections due to *S. maltophilia* resistant to cotrimoxazole, cefiderocol and the combination of ceftazidime–avibactam and aztreonam are promising options; however, further clinical data are needed to evaluate their efficacy.

## 4. Conclusions

Carbapenem-resistant Gram-negative bacteria represent a serious threat for public health due to the high burden of morbidity and mortality and the limited treatment options. Novel agents have been developed in recent years against these pathogens, and several others are in the pipeline. Considering that the different molecules are active against specific mechanisms of resistance, the molecular characterization of the isolates is becoming of utmost importance for a correct therapeutic management of patients. Further data are needed to define the most appropriate role of the newly available antibiotic combinations.

## Figures and Tables

**Table 1:** The five Ds of antimicrobial stewardship.

**D**iagnosis	Make the right diagnosis based on signs and symptoms and laboratory findings
**D**rug	In the context of empiric treatment, always choose the drug on the base of international guidelines and local epidemiology
**D**ose	Always use the appropriate dose, avoiding underdosage especially in critical ill setting. Always adjust the dosage in accordance with hepatic and renal impairment
**D**uration	Pay attention to the right duration of antibiotic treatment. Avoid the “longer is better” mentality
**D**e-escalation	Based on the antimicrobial susceptibility tests results, always de-escalate therapy, preferring monotherapy when possible.

**Table 2:** Major carbapenemase enzymes. Adapted from: Tompkins K et al. [8].

Ambler Class	Major Enzymes	Active Site	Treatment Notes
A	KPC, NMC, SME	Serine	Clavulanate and tazobactam are not effective Avibactam, vaborbactam and relebactam are effective
B	VIM, IMP, NDM	Zinc	All available BLI are ineffective Do not hydrolyze monobactams and cefiderocol
D	OXA-48	Serine	Clavulanate, tazobactam, vaborbactam and relebactam are not effective Avibactam is effective

KPC: K. pneumoniae Carbapenemase; NMC: not metalloenzyme carbapenemase; SME: Serratia marcescens enzyme; VIM: Verona Integron-encoded metallo-beta-lactamase; IMP: Imipenemase; NDM: Delhi metallo-β-lactamase; OXA: oxacillinase.

**Table 3:** Key recommendations from different recent authoritative guidelines on DTR-PA infections.

IDSA [12]	ESCMID [13]	Italian Guidelines [14]
**General approach**		
Ceftolozane–tazobactam, ceftazidime–avibactam, imipenem–cilastatin–relebactam and cefiderocol as monotherapy are preferred options for the treatment of infections outside of the urinary tract and of the urinary tract; a single dose of an aminoglycoside or cefiderocol may also be used for uncomplicated cystitis; cefiderocol may be used for pyelonephritis and complicated urinary tract infections as well.	Therapy with ceftolozane–tazobactam, if active in vitro, is suggested (*conditional recommendation*, *very low certainty of evidence*). Insufficient evidence is available for imipenem–relebactam, cefiderocol and ceftazidime–avibactam.	Ceftolozane/tazobactam and ceftazidime/avibactam are deemed the first-line options for targeted treatment. Imipenem/cilastatin– relebactam and cefiderocol might be potential alternatives, as well as colistin-based therapy (*strong recommendation*, *moderate certainity of evidence*).
**Monotherapy versus combination therapy**		
Combination antibiotic therapy is not routinely recommended if in vitro susceptibility to a first-line antibiotic (i.e., ceftolozane–tazobactam, ceftazidime–avibactam, or imipenem–cilastatin–relebactam) has been confirmed.	Lacking evidence, no recommendation can be made for or against the use of combination therapy with the new beta-lactams (ceftazidime–avibactam and ceftolozane–tazobactam). When treating severe infections caused by CRPA with polymyxins, aminoglycosides or fosfomycin, a treatment with two in vitro active drugs is suggested (*conditional recommendation*, *very low certainty of evidence*). No recommendation for or against specific combinations can be made.	Combination therapy should not be the routine choice but may be considered on a case-by-case basis, especially upon consultation with infectious diseases specialists. In particular, combination regimens including fosfomycin as companion agent could be considered (*conditional recommendation*, *low certainity of evidence*).

## References

[1] European Centre for Disease Prevention and Control; Antimicrobial Resistance Surveillance in Europe 2022–2020 Data. https://www.ecdc.europa.eu/en/publications-data/antimicrobial-resistance-surveillance-europe-2022-2020-data.

[2] World Health Organization Global Action Plan on Antimicrobial Resistance. WHO 2015. https://www.who.int/publications/i/item/9789241509763.

[3] Saini V., Jain C., Singh N.P., Alsulimani A., Gupta C., Dar S.A., Haque S., Das S. (2021). Paradigm shift in antimicrobial resistance pattern of bacterial isolates during the COVID-19 pandemic. Antibiotics.

[4] European Centre for Disease Control Carbapenem-Resistant Enterobacteriaceae-Second Update; 2019. https://www.ecdc.europa.eu/sites/default/files/documents/carbapenem-resistant-enterobacteriaceae-risk-assessment-rev-2.pdf.

[5] European Centre for Disease Control Surveillance of Antimicrobial Resistance in Europe–Annual Report of the European Antimicrobial Resistance Surveillance Network (EARS-Net); 2018. https://www.ecdc.europa.eu/sites/default/files/documents/AMR%202017_Cover%2BInner-web_v3.pdf.

[6] Ruppé É., Woerther P.L., Barbier F. (2015). Mechanisms of antimicrobial resistance in Gram-negative bacilli. Ann. Intensive Care.

[7] Kazmierczak K.M., Karlowsky J.A., de Jonge B.L.M., Stone G.G., Sahm D.F. (2021). Epidemiology of carbapenem resistance determinants identified in meropenem-nonsusceptible enterobacterales collected as part of a global surveillance program, 2012 to 2017. Antimicrob. Agents Chemother..

[8] Tompkins K., van Duin D. (2021). Treatment for carbapenem-resistant Enterobacterales infections: Recent advances and future directions. Eur. J. Clin. Microbiol. Infect. Dis..

[9] Behzadi P., Baráth Z., Gajdács M. (2021). It’s not easy being green: A narrative review on the microbiology, virulence and therapeutic prospects of multidrug-resistant pseudomonas aeruginosa. Antibiotics.

[10] Bedos J.P., Daikos G., Dodgson A.R., Pan A., Petrosillo N., Seifert H., Vila J., Ferrer R., Wilson P. (2021). Early identification and optimal management of carbapenem-resistant Gram-negative infection. J. Hosp. Infect..

[11] Kadri S.S., Adjemian J., Lai Y.L., Spaulding A.B., Ricotta E., Rebecca Prevots D., Palmore T.N., Rhee C., Klompas M., Dekker J.P. (2018). Difficult-to-treat resistance in gram-negative bacteremia at 173 US hospitals: Retrospective cohort analysis of prevalence, predictors, and outcome of resistance to all first-line agents. Clin. Infect. Dis..

[12] Tamma P.D., Aitken S.L., Bonomo R.A., Mathers A.J., Van Duin D., Clancy C.J. (2022). Infectious Diseases Society of America Guidance on the Treatment of Extended-Spectrum β-lactamase Producing Enterobacterales (ESBL-E), Carbapenem-Resistant Enterobacterales (CRE), and Pseudomonas aeruginosa with Difficult-to-Treat Resistance (DTR-P. aerug). Clin. Infect. Dis..

[13] Paul M., Carrara E., Retamar P., Tängdén T., Bitterman R., Bonomo R.A., de Waele J., Daikos G.L., Akova M., Harbarth S. (2022). European Society of Clinical Microbiology and Infectious Diseases (ESCMID) guidelines for the treatment of infections caused by multidrug-resistant Gram-negative bacilli (endorsed by European society of intensive care medicine). Clin. Microbiol. Infect..

[14] Tiseo G., Brigante G., Giacobbe D.R., Maraolo A.E., Gona F., Falcone M., Giannella M., Grossi P., Pea F., Rossolini G.M. (2022). Diagnosis and management of infections caused by multidrug-resistant bacteria: Guideline endorsed by the Italian Society of Infection and Tropical Diseases (SIMIT), the Italian Society of Anti-Infective Therapy (SITA), the Italian Group for Antimicrobial Stewardship (GISA), the Italian Association of Clinical Microbiologists (AMCLI) and the Italian Society of Microbiology (SIM). Int. J. Antimicrob. Agents.

[15] Al-Anazi K.A., Al-Jasser A.M. (2014). Infections caused by Acinetobacter baumannii in recipients of ematopoietic stem cell transplantation. Front. Oncol..

[16] Abdul-Mutakabbir J.C., Griffith N.C., Shields R.K., Tverdek F.P., Escobar Z.K. (2021). Contemporary Perspective on the Treatment of Acinetobacter baumannii Infections: Insights from the Society of Infectious Diseases Pharmacists. Infect. Dis. Ther..

[17] Hujer K.M., Hamza N.S., Hujer A.M., Perez F., Helfand M.S., Bethel C.R., Thomson J.M., Anderson V.E., Barlow M., Rice L.B. (2005). Identification of a new allelic variant of the Acinetobacter baumannii cephalosporinase, ADC-7 β-lactamase: Defining a unique family of class C enzymes. Antimicrob. Agents Chemother..

[18] Higgins P.G., Poirel L., Lehmann M., Nordmann P., Seifert H. (2009). OXA-143, a novel carbapenem-hydrolyzing class D β-lactamase in Acinetobacter baumannii. Antimicrob. Agents Chemother..

[19] Higgins P.G., Pérez-Llarena F.J., Zander E., Fernández A., Bou G., Seifert H. (2013). OXA-235, a novel class D β-lactamase involved in resistance to carbapenems in Acinetobacter baumannii. Antimicrob. Agents Chemother..

[20] Poirel L., Pitout J.D., Nordmann P. (2007). Carbapenemases: Molecular diversity and clinical consequences. Future Microbiol..

[21] Manchanda V., Sinha S., Singh N. (2010). Multidrug resistant Acinetobacter. J. Glob. Infect. Dis..

[22] Li J., Nation R.L., Milne R.W., Turnidge J.D., Coulthard K. (2005). Evaluation of colistin as an agent against multi-resistant Gram-negative bacteria. Int. J. Antimicrob. Agents.

[23] Giamarellou H., Antoniadou A., Kanellakopoulou K. (2008). Acinetobacter baumannii: A universal threat to public health?. Int. J. Antimicrob. Agents.

[24] Sader H.S., Castanheira M., Mendes R.E., Flamm R.K. (2018). Frequency and antimicrobial susceptibility of Gram-negative bacteria isolated from patients with pneumonia hospitalized in ICUs of US medical centres (2015–17). J. Antimicrob. Chemother..

[25] Cai B., Tillotson G., Benjumea D., Callahan P., Echols R. (2020). The burden of bloodstream infections due to stenotrophomonas maltophilia in the United States: A large, retrospective database study. Open Forum Infect. Dis..

[26] Brooke J.S. (2021). Advances in the microbiology of stenotrophomonas maltophilia. Clin. Microbiol. Rev..

[27] Sánchez M.B. (2015). Antibiotic resistance in the opportunistic pathogen Stenotrophomonas maltophilia. Front. Microbiol..

[28] Gentile I., Maraolo A.E., Borgia G. (2016). What is the role of the new β-lactam/β-lactamase inhibitors ceftolozane/tazobactam and ceftazidime/avibactam?. Expert Rev. Anti. Infect. Ther..

[29] Solomkin J., Hershberger E., Miller B., Popejoy M., Friedland I., Steenbergen J., Yoon M., Collins S., Yuan G., Barie P.S. (2015). Ceftolozane/tazobactam plus metronidazole for complicated intra-abdominal infections in an era of multidrug resistance: Results from a randomized, double-blind, phase 3 trial (ASPECT-cIAI). Clin. Infect. Dis..

[30] Wagenlehner F.M., Umeh O., Steenbergen J., Yuan G., Darouiche R.O. (2015). Ceftolozane-tazobactam compared with levofloxacin in the treatment of complicated urinary-tract infections, including pyelonephritis: A randomised, double-blind, phase 3 trial (ASPECT-cUTI). Lancet.

[31] Kollef M.H., Nováček M., Kivistik Ü., Réa-Neto Á., Shime N., Martin-Loeches I., Timsit J.F., Wunderink R.G., Bruno C.J., Huntington J.A. (2019). Ceftolozane–tazobactam versus meropenem for treatment of nosocomial pneumonia (ASPECT-NP): A randomised, controlled, double-blind, phase 3, non-inferiority trial. Lancet Infect. Dis..

[32] Yahav D., Giske C.G., Gramatniece A., Abodakpi H., Tam V.H., Leibovici L. (2021). New β-lactam–β-lactamase inhibitor combinations. Clin. Microbiol. Rev..

[33] Farrell. D.J., Flamm R.K., Sader H.S., Jones R.N. (2013). Farrell. D.J.; Flamm, R.K.; Sader, H.S.; Jones, R.N. Antimicrobial activity of ceftolozane-tazobactam tested against Enterobacteriaceae and Pseudomonas aeruginosa with various resistance patterns isolated in U.S. hospitals (2011–2012). Antimicrob. Agents Chemother..

[34] Sutherland C.A., Nicolau D.P. (2015). Susceptibility profile of ceftolozane/tazobactam and other parenteral antimicrobials against *Escherichia coli*, *Klebsiella pneumoniae*, and *Pseudomonas aeruginosa* from US hospitals. Clin. Ther..

[35] Carvalhaes C.G., Castanheira M., Sader H.S., Flamm R.K., Shortridge D. (2019). Antimicrobial activity of ceftolozane–tazobactam tested against gram-negative contemporary (2015–2017) isolates from hospitalized patients with pneumonia in US medical centers. Diagn. Microbiol. Infect. Dis..

[36] Puzniak L., Dillon R., Palmer T., Collings H., Enstone A. (2021). Real-world use of ceftolozane/tazobactam: A systematic literature review. Antimicrob. Resist. Infect. Control..

[37] Pogue J.M., Kaye K.S., Veve M.P., Patel T.S., Gerlach A.T., Davis S.L., Puzniak L.A., File T.M., Olson S., Dhar S. (2020). Ceftolozane/tazobactam vs polymyxin or aminoglycoside-based regimens for the treatment of drug-resistant Pseudomonas aeruginosa. Clin. Infect. Dis..

[38] Haidar G., Philips N.J., Shields R.K., Snyder D., Cheng S., Potoski B.A., Doi Y., Hao B., Press E.G., Cooper V.S. (2017). Ceftolozane-Tazobactam for the Treatment of Multidrug-Resistant Pseudomonas aeruginosa Infections: Clinical Effectiveness and Evolution of Resistance. Clin. Infect. Dis..

[39] Tamma P.D., Beisken S., Bergman Y., Posch A.E., Avdic E., Sharara S.L., Cosgrove S.E., Simner P.J. (2021). Modifiable Risk Factors for the Emergence of Ceftolozane-tazobactam Resistance. Clin. Infect. Dis..

[40] Bassetti M., Vena A., Giacobbe D.R., Falcone M., Tiseo G., Giannella M., Pascale R., Meschiari M., Digaetano M., Oliva A. (2020). Ceftolozane/tazobactam for treatment of severe ESBL-producing enterobacterales infections: A multicenter nationwide clinical experience (CEFTABUSE II Study). Open Forum Infect. Dis..

[41] Corcione S., Lupia T., Maraolo A.E., Mornese Pinna S., Gentile I., De Rosa F.G. (2019). Carbapenem-sparing strategy: Carbapenemase, treatment, and stewardship. Curr. Opin. Infect. Dis..

[42] U.S. Food and Drug Administration 2018. NDA Multi-Disciplinary Review and Evaluation—NDA 206494 Supplements 005 and 006 AVYCAZ (Ceftazidime/Avibactam) for Injection. https://www.fda.gov/media/124307/download.

[43] European Medicines Agency (2020). An Overview of Zavicefta and Why It Is Authorised in the EU. https://www.ema.europa.eu/en/documents/overview/zavicefta-epar-medicine-overview_en.pdf.

[44] O’Callaghan H. (1986). Ceftazidime, a broad spectrum cephalosporin with activity against Ps. aeruginosa. J. Hyg. Epidemiol. Microbiol. Immunol.

[45] Coleman K. (2011). Diazabicyclooctanes (DBOs): A potent new class of non-β-lactam β-lactamase inhibitors. Curr. Opin. Microbiol..

[46] Ehmann D.E., Jahić H., Ross P.L., Gu R.F., Hu J., Durand-Réville T.F., Lahiri S., Thresher J., Livchak S., Gao N. (2013). Kinetics of avibactam inhibition against class A, C, and D β-lactamases. J. Biol. Chem..

[47] van Duin D., Bonomo R.A. (2021). Ceftazidime/avibactam and ceftolozane/tazobactam: Second-generation β-Lactam/β-lactamase inhibitor combinations. Clin. Infect. Dis..

[48] Liscio J.L., Mahoney M.V., Hirsch E.B. (2015). Ceftolozane/tazobactam and ceftazidime/avibactam: Two novel β-lactam/β-lactamase inhibitor combination agents for the treatment of resistant Gram-negative bacterial infections. Int. J. Antimicrob. Agents.

[49] Biedenbach D.J., Kazmierczak K., Bouchillon S.K., Sahm D.F., Bradford P.A. (2015). In vitro activity of aztreonam-avibactam against a global collection of Gram-negative pathogens from 2012 and 2013. Antimicrob. Agents Chemother..

[50] Mushtaq S., Warner M., Williams G., Critchley I., Livermore D.M. (2010). Activity of chequerboard combinations of ceftaroline and NXL104 versus β-lactamase-producing Enterobacteriaceae. J. Antimicrob. Chemother..

[51] Aktaş Z., Kayacan C., Oncul O. (2012). In vitro activity of avibactam (NXL104) in combination with β-lactams against Gram-negative bacteria, including OXA-48 β-lactamase-producing Klebsiella pneumoniae. Int. J. Antimicrob. Agents.

[52] Haidar G., Clancy C.J., Shields R.K., Hao B., Cheng S., Nguyen M.H. (2017). Mutations in blaKPC-3 that confer ceftazidime-avibactam resistance encode novel KPC-3 variants that function as extended-spectrum β-lactamases. Antimicrob. Agents Chemother..

[53] Nelson K., Hemarajata P., Sun D., Rubio-Aparicio D., Tsivkovski R., Yang S., Sebra R., Kasarskis A., Nguyen H., Hanson B.M. (2017). Resistance to ceftazidime-avibactam is due to transposition of KPC in a porin-deficient strain of Klebsiella pneumoniae with increased efflux activity. Antimicrob. Agents Chemother..

[54] Chalhoub H., Sáenz Y., Nichols W.W., Tulkens P.M., Van Bambeke F. (2018). Loss of activity of ceftazidime-avibactam due to MexAB-OprM efflux and overproduction of AmpC cephalosporinase in Pseudomonas aeruginosa isolated from patients suffering from cystic fibrosis. Int. J. Antimicrob. Agents.

[55] Sanz-García F., Hernando-Amado S., Martínez J.L. (2018). Mutation-driven evolution of pseudomonas aeruginosa in the presence of either ceftazidime or ceftazidime-avibactam. Antimicrob. Agents Chemother..

[56] Kazmierczak K.M., Bradford P.A., Stone G.G., De Jonge B.L.M., Sahm D.F. (2018). In vitro activity of ceftazidime-avibactam and aztreonam-avibactam against OXA-48-carrying Enterobacteriaceae isolated as part of the International Network for Optimal Resistance Monitoring (INFORM) global surveillance program from 2012 to 2015. Antimicrob. Agents Chemother..

[57] Mazuski J.E., Gasink L.B., Armstrong J., Broadhurst H., Stone G.G., Rank D., Llorens L., Newell P., Pachl J. (2016). Efficacy and safety of ceftazidime-avibactam plus metronidazole versus meropenem in the treatment of complicated intra-abdominal infection: Results from a randomized, controlled, double-blind, phase 3 program. Clin. Infect. Dis..

[58] Carmeli Y., Armstrong J., Laud P.J., Newell P., Stone G., Wardman A., Gasink L.B. (2016). Ceftazidime-avibactam or best available therapy in patients with ceftazidime-resistant Enterobacteriaceae and Pseudomonas aeruginosa complicated urinary tract infections or complicated intra-abdominal infections (REPRISE): A randomised, pathogen-directed, phase 3 study. Lancet Infect. Dis..

[59] Wagenlehner F.M., Sobel J.D., Newell P., Armstrong J., Huang X., Stone G.G., Yates K., Gasink L.B. (2016). Ceftazidime-avibactam Versus Doripenem for the Treatment of Complicated Urinary Tract Infections, Including Acute Pyelonephritis: RECAPTURE, a Phase 3 Randomized Trial Program. Clin. Infect. Dis..

[60] Torres A., Zhong N., Pachl J., Timsit J.F., Kollef M., Chen Z., Song J., Taylor D., Laud P.J., Stone G.G. (2018). Ceftazidime-avibactam versus meropenem in nosocomial pneumonia, including ventilator-associated pneumonia (REPROVE): A randomised, double-blind, phase 3 non-inferiority trial. Lancet Infect. Dis..

[61] Tumbarello M., Raffaelli F., Giannella M., Mantengoli E., Mularoni A., Venditti M., De Rosa F.G., Sarmati L., Bassetti M., Brindicci G. (2021). Ceftazidime-avibactam use for klebsiella pneumoniae carbapenemase-producing k. pneumoniae infections: A retrospective observational multicenter study. Clin. Infect. Dis..

[62] Onorato L., Di Caprio G., Signoriello S., Coppola N. (2019). Efficacy of ceftazidime/avibactam in monotherapy or combination therapy against carbapenem-resistant Gram-negative bacteria: A meta-analysis. Int. J. Antimicrob. Agents.

[63] Vabomere Highlights of Prescribing Information. https://www.accessdata.fda.gov/drugsatfda_docs/label/2017/209776lbl.pdf.

[64] EMA Assessment Report-Vabomere. https://www.ema.europa.eu/en/documents/assessment-report/vabomere-epar-public-assessment-report_en.pdf.

[65] Pascale R., Giannella M., Bartoletti M., Viale P., Pea F. (2019). Use of meropenem in treating carbapenem-resistant Enterobacteriaceae infections. Expert Rev. Anti. Infect. Ther..

[66] Hecker S.J., Reddy K.R., Totrov M., Hirst G.C., Lomovskaya O., Griffith D.C., King P., Tsivkovski R., Sun D., Sabet M. (2015). Discovery of a cyclic boronic acid β-lactamase inhibitor (RPX7009) with utility vs class A serine carbapenemases. J. Med. Chem..

[67] Hackel M.A., Lomovskaya O., Dudley M.N., Karlowsky J.A., Sahm D.F. (2018). In vitro activity of meropenem-vaborbactam against clinical isolates of KPC-positive Enterobacteriaceae. Antimicrob. Agents Chemother..

[68] Shortridge D., Carvalhaes C., Deshpande L., Castanheira M. (2021). Activity of meropenem/vaborbactam and comparators against Gram-negative isolates from Eastern and Western European patients hospitalized with pneumonia including ventilator-associated pneumonia (2014-19). J. Antimicrob. Chemother..

[69] Savov E., Trifonova A., Kovachka K., Kjosseva E., Strateva T. (2019). Antimicrobial in vitro activities of ceftazidime-avibactam, meropenem-vaborbactam and plazomicin against multidrug-resistant Acinetobacter baumannii and Pseudomonas aeruginosa–a pilot Bulgarian study. Infect. Dis..

[70] Zhanel G.G., Lawrence C.K., Adam H., Schweizer F., Zelenitsky S., Zhanel M., Lagacé-Wiens P.R.S., Walkty A., Denisuik A., Golden A. (2018). Imipenem–Relebactam and Meropenem–Vaborbactam: Two Novel Carbapenem-β-Lactamase Inhibitor Combinations. Drugs.

[71] Kaye K.S., Bhowmick T., Metallidis S., Bleasdale S.C., Sagan O.S., Stus V., Vazquez J., Zaitsev V., Bidair M., Chorvat E. (2018). Effect of meropenem-vaborbactam vs. piperacillin-Tazobactam on clinical cure or improvement and microbial eradication in complicated urinary tract infection the TANGO I randomized clinical trial. JAMA.

[72] Wunderink R.G., Giamarellos-Bourboulis E.J., Rahav G., Mathers A.J., Bassetti M., Vazquez J., Cornely O.A., Solomkin J., Bhowmick T., Bishara J. (2018). Effect and Safety of Meropenem–Vaborbactam versus Best-Available Therapy in Patients with Carbapenem-Resistant Enterobacteriaceae Infections: The TANGO II Randomized Clinical Trial. Infect. Dis. Ther..

[73] Alosaimy S., Lagnf A.M., Morrisette T., Scipione M.R., Zhao J.J., Jorgensen S.C.J., Mynatt R., Carlson T.J., Jo J., Garey K.W. (2021). Real-world, Multicenter Experience with Meropenem-Vaborbactam for Gram-Negative Bacterial Infections including Carbapenem-Resistant Enterobacterales and Pseudomonas aeruginosa. Open Forum Infect. Dis..

[74] Ackley R., Roshdy D., Meredith J., Minor S., Anderson W.E., Capraro G.A., Polk C. (2020). Meropenem-vaborbactam versus ceftazidime-avibactam for treatment of carbapenem-resistant enterobacteriaceae infections. Antimicrob. Agents Chemother..

[75] Heo Y.A. (2021). Imipenem/Cilastatin/Relebactam: A Review in Gram-Negative Bacterial Infections. Drugs.

[76] Principe L., Lupia T., Andriani L., Campanile F., Carcione D., Corcione S., De Rosa F.G., Luzzati R., Stroffolini G., Steyde M. (2022). Microbiological, Clinical, and PK/PD Features of the New Anti-Gram-Negative Antibiotics: β-Lactam/β-Lactamase Inhibitors in Combination and Cefiderocol—An All-Inclusive Guide for Clinicians. Pharmaceuticals.

[77] Motsch J., De Oliveira C.U.M., Stus V., Kö Ksal I., Lyulko O., Boucher H.W., Kaye K.S., File T.M., Brown M.L., Khan I. (2020). RESTORE-IMI 1: A Multicenter, Randomized, Doubleblind Trial Comparing Efficacy and Safety of Imipenem/Relebactam vs Colistin Plus Imipenem in Patients with Imipenem-nonsusceptible Bacterial Infections. Clin. Infect. Dis..

[78] Titov I., Wunderink R.G., Roquilly A., Gonzalez D.R., David-Wang A., Boucher H.W., Kaye K.S., Losada M.C., Du J., Tipping R. (2021). A Randomized, Double-blind, Multicenter Trial Comparing Efficacy and Safety of Imipenem/Cilastatin/Relebactam Versus Piperacillin/Tazobactam in Adults with Hospital-acquired or Ventilator-associated Bacterial Pneumonia (RESTORE-IMI 2 Study). Clin. Infect. Dis..

[79] Brown M.L., Motsch J., Kaye K.S., File T.M., Boucher H.W., Vendetti N., Aggrey A., Joeng H.K., Tipping R.W., Du J. (2020). Evaluation of renal safety between imipenem/relebactam and colistin plus imipenem in patients with imipenem-nonsusceptible bacterial infections in the randomized, Phase 3 RESTORE-IMI 1 Study. Open Forum. Infect. Dis..

[80] Rebold N., Morrisette T., Lagnf A.M., Alosaimy S., Holger D., Barber K., Justo J.A., Antosz K., Carlson T.J., Frens J.J. (2021). Early Multicenter Experience with Imipenem-Cilastatin-Relebactam for Multidrug-Resistant Gram-Negative Infections. Open Forum Infect. Dis..

[81] Balabanian G., Rose M., Manning N., Landman D., Quale J. (2018). Effect of porins and blaKPC expression on activity of imipenem with relebactam in klebsiella pneumoniae: Can antibiotic combinations overcome resistance?. Microb. Drug. Resist..

[82] McCreary E.K., Heil E.L., Tamma P.D. (2021). New perspectives on antimicrobial agents: Cefiderocol. Antimicrob. Agents Chemother..

[83] Ito A., Sato T., Ota M., Takemura M., Nishikawa T., Toba S., Kohira N., Miyagawa S., Ishibashi N., Matsumoto S. (2018). In vitro antibacterial properties of cefiderocol, a novel siderophore cephalosporin, against gram-negative bacteria. Antimicrob. Agents Chemother..

[84] Ong’Uti S., Czech M., Robilotti E., Holubar M. (2022). Cefiderocol: A New Cephalosporin Stratagem Against Multidrug-Resistant Gram-Negative Bacteria. Clin. Infect. Dis..

[85] Sato T., Yamawaki K. (2019). Cefiderocol: Discovery, Chemistry, and in Vivo Profiles of a Novel Siderophore Cephalosporin. Clin. Infect. Dis..

[86] Abdul-Mutakabbir J.C., Alosaimy S., Morrisette T., Kebriaei R., Rybak M.J. (2020). Cefiderocol: A Novel Siderophore Cephalosporin against Multidrug-Resistant Gram-Negative Pathogens. Pharmacotherapy.

[87] Hackel M.A., Tsuji M., Yamano Y., Echols R., Karlowsky J.A., Sahma D.F. (2017). In vitro activity of the siderophore cephalosporin, cefiderocol, against a recent collection of clinically relevant gram-negative Bacilli from North America and Europe, including carbapenem-nonsusceptible isolates (SIDERO-WT-2014 study). Antimicrob. Agents Chemother..

[88] Kazmierczak K.M., Tsuji M., Wise M.G., Hackel M., Yamano Y., Echols R., Sahm D.F. (2019). In vitro activity of cefiderocol, a siderophore cephalosporin, against a recent collection of clinically relevant carbapenem-non-susceptible Gram-negative bacilli, including serine carbapenemase- and metallo-β-lactamase-producing isolates (SIDERO-WT-2014 Study). Int. J. Antimicrob. Agents.

[89] Longshaw C., Manissero D., Tsuji M., Echols R., Yamano Y. (2020). In vitro activity of the siderophore cephalosporin, cefiderocol, against molecularly characterized, carbapenem-non-susceptible Gram-negative bacteria from Europe. JAC Antimicrob. Resist..

[90] Wang C., Yang D., Wang Y., Ni W. (2022). Cefiderocol for the Treatment of Multidrug-Resistant Gram-Negative Bacteria: A Systematic Review of Currently Available Evidence. Front. Pharmacol..

[91] Nurjadi D., Kocer K., Chanthalangsy Q., Klein S., Heeg K., Boutin S. (2022). New Delhi Metallo-Beta-Lactamase Facilitates the Emergence of Cefiderocol Resistance in Enterobacter cloacae. Antimicrob. Agents Chemother..

[92] Karakonstantis S., Rousaki M., Kritsotakis E.I. (2022). Cefiderocol: Systematic Review of Mechanisms of Resistance, Heteroresistance and In Vivo Emergence of Resistance. Antibiotics.

[93] Matsumoto S., Singley C.M., Hoover J., Nakamura R., Echols R., Rittenhouse S., Tsuji M., Yamano Y. (2017). Efficacy of cefiderocol against carbapenem-resistant gram-negative bacilli in immunocompetent-rat respiratory tract infection models recreating human plasma pharmacokinetics. Antimicrob. Agents Chemother..

[94] El-Sayed Ahmed M.A.E.G., Zhong L.L., Shen C., Yang Y., Doi Y., Tian G.B. (2020). Colistin and its role in the Era of antibiotic resistance: An extended review (2000–2019). Emerg. Microbes Infect..

[95] Dijkmans A.C., Wilms E.B., Kamerling I.M.C., Birkhoff W., Ortiz-Zacarías N.V., Van Nieuwkoop C., Verbrugh H.A., Touw D.J. (2015). Colistin: Revival of an Old Polymyxin Antibiotic. Ther. Drug Monit..

[96] Li J., Nation R.L., Turnidge J.D., Milne R.W., Coulthard K., Rayner C.R., Paterson D.L. (2006). Colistin: The re-emerging antibiotic for multidrug-resistant Gram-negative bacterial infections. Lancet Infect. Dis..

[97] Tsuji B.T., Pogue J.M., Zavascki A.P., Paul M., Daikos G.L., Forrest A., Giacobbe D.R., Viscoli C., Giamarellou H., Karaiskos I. (2019). International Consensus Guidelines for the Optimal Use of the Polymyxins: Endorsed by the American College of Clinical Pharmacy (ACCP), European Society of Clinical Microbiology and Infectious Diseases (ESCMID), Infectious Diseases Society of America (IDSA), International Society for Anti-infective Pharmacology (ISAP), Society of Critical Care Medicine (SCCM), and Society of Infectious Diseases Pharmacists (SIDP). Pharmacotherapy.

[98] Castanheira M., Griffin M.A., Deshpande L.M., Mendes R.E., Jones R.N., Flamm R.K. (2016). Detection of mcr-1 among Escherichia coli clinical isolates collected worldwide as part of the SENTRY Antimicrobial Surveillance Program in 2014 and 2015. Antimicrob. Agents Chemother..

[99] Bastidas-Caldes C., de Waard J.H., Salgado M.S., Villacís M.J., Coral-Almeida M., Yamamoto Y., Calvopiña M. (2022). Worldwide Prevalence of mcr-mediated Colistin-Resistance Escherichia coli in Isolates of Clinical Samples, Healthy Humans, and Livestock-A Systematic Review and Meta-Analysis. Pathogens.

[100] Falagas M.E., Vouloumanou E.K., Samonis G., Vardakas K.Z. (2016). Fosfomycin. Clin. Microbiol. Rev..

[101] Anderson G.G., Kenney T.F., Macleod D.L., Henig N.R., O’Toole G.A. (2013). Eradication of Pseudomonas aeruginosa biofilms on cultured airway cells by a fosfomycin/tobramycin antibiotic combination. Pathog. Dis..

[102] Barry A.L., Brown S.D. (1995). Antibacterial spectrum of fosfomycin trometamol. J. Antimicrob. Chemother..

[103] Patel S.S., Balfour J.A., Bryson H.M. (1997). Fosfomycin Tromethamine. A review of its antibacterial activity, pharmacokinetic properties and therapeutic efficacy as a single-dose oral treatment for acute uncomplicated lower urinary tract infections. Drugs.

[104] Samonis G., Maraki S., Rafailidis P.I., Kapaskelis A., Kastoris A.C., Falagas M.E. (2010). Antimicrobial susceptibility of Gram-negative nonurinary bacteria to fosfomycin and other antimicrobials. Future Microbiol..

[105] De Smet K.A.L., Kempsell K.E., Gallagher A., Duncan K., Young D.B. (1999). Alteration of a single amino acid residue reverses fosfomycin resistance of recombinant MurA from Mycobacterium tuberculosis. Microbiology.

[106] CLSI Clinical and Laboratory Standards Institute Performance Standards for Antimicrobial Susceptibility Testing; Twenty-Fifth Informational Supplement. CLSI Document M100-S25; 2015; Vol. 32. https://clsi.org/standards/products/microbiology/documents/m100/.

[107] Boyanova L. (2015). Susceptibility of anaerobes to fusidic acid and fosfomycin. Int. J. Antimicrob. Agents.

[108] Mikhail S., Singh N.B., Kebriaei R., Rice S.A., Stamper K.C., Castanheira M., Rybak M.J. (2019). Evaluation of the synergy of ceftazidime-avibactam in combination with meropenem, amikacin, aztreonam, colistin, or fosfomycin against well-characterized multidrug-resistant klebsiella pneumoniae and pseudomonas aeruginosa. Antimicrob. Agents Chemother..

[109] MacLeod D.L., Barker L.M., Sutherland J.L., Moss S.C., Gurgel J.L., Kenney T.F., Burns J.L., Baker W.R. (2009). Antibacterial activities of a fosfomycin/tobramycin combination: A novel inhaled antibiotic for bronchiectasis. J. Antimicrob. Chemother..

[110] Mazzei T., Cassetta M.I., Fallani S., Arrigucci S., Novelli A. (2006). Pharmacokinetic and pharmacodynamic aspects of antimicrobial agents for the treatment of uncomplicated urinary tract infections. Int. J. Antimicrob. Agents.

[111] Krause K.M., Serio A.W., Kane T.R., Connolly L.E. (2016). Aminoglycosides: An overview. Cold Spring Harb. Perspect. Med..

[112] Eljaaly K., Alharbi A., Alshehri S., Ortwine J.K., Pogue J.M. (2019). Plazomicin: A Novel Aminoglycoside for the Treatment of Resistant Gram-Negative Bacterial Infections. Drugs.

[113] Wagenlehner F.M.E., Cloutier D.J., Komirenko A.S., Cebrik D.S., Krause K.M., Keepers T.R., Connolly L.E., Miller L.G., Friedland I., Dwyer J.P. (2019). Re: Once-daily plazomicin for complicated urinary tract infections. NEJM.

[114] Connolly L.E., Riddle V., Cebrik D., Armstrong E.S., Miller L.G. (2018). A multicenter, randomized, double-blind, phase 2 study of the efficacy and safety of plazomicin compared with levofloxacin in the treatment of complicated urinary tract infection and acute pyelonephritis. Antimicrob. Agents Chemother..

[115] Yusuf E., Bax H.I., Verkaik N.J., van Westreenen M. (2021). An update on eight “new” antibiotics against multidrug-resistant gram-negative bacteria. J. Clin. Med..

[116] McKinnell J.A., Dwyer J.P., Talbot G.H., Connolly L.E., Friedland I., Smith A., Jubb A.M., Serio A.W., Krause K.M., Daikos G.L. (2019). Plazomicin for Infections Caused by Carbapenem-Resistant Enterobacteriaceae. N. Engl. J. Med..

[117] Tang H.J., Lai C.C. (2020). Plazomicin-associated Nephrotoxicity. Clin. Infect. Dis..

[118] Chou A., Welch E., Hunter A., Trautner B.W. (2022). Antimicrobial Treatment Options for Difficult-to-Treat Resistant Gram-Negative Bacteria Causing Cystitis, Pyelonephritis, and Prostatitis: A Narrative Review. Drugs.

[119] EUCTR2018-002526-23-LT. A Clinical Trial to Evaluate the Efficacy and Safety of Sulbactam-ETX2514, A New Drug in the Treatment of Patients with ABC Infections Caused by Acinetobacter baumannii-calcoaceticus Complex. http://www.who.int/trialsearch/Trial2.aspx?TrialID=EUCTR2018-002526-23-LT.

[120] Isler B., Harris P., Stewart A.G., Paterson D.L. (2021). An update on cefepime and its future role in combination with novel β-lactamase inhibitors for MDR Enterobacterales and Pseudomonas aeruginosa. J. Antimicrob. Chemother..

[121] Bansal N., Sukhwani K.S., Kumar D.S., Nambi P.S., Gopalakrishnan R., Ramasubramanian V. (2018). Clinical efficacy and safety of cefepime–tazobactam in hospitalized patients in South India. Infect. Dis..

[122] Bajaksouzian S., Rutter J.D., Reghal A., Shapiro S., Taracila M.A., Jacobs M.R., Bonomo R.A., Jacqueline C., Papp-Wallace K.M., Bethel C.R. (2019). Beyond piperacillin-tazobactam: Cefepime and AAI101 as a potent -lactam-lactamase inhibitor combination. Antimicrob. Agents Chemother..

[123] Bassetti M., Vena A., Sepulcri C., Giacobbe D.R., Peghin M. (2020). Treatment of bloodstream infections due to gram-negative bacteria with difficult-to-treat resistance. Antibiotics.

[124] Karaiskos I., Lagou S., Pontikis K., Rapti V., Poulakou G. (2019). The “Old” and the “New” antibiotics for MDR Gram-negative pathogens: For whom, when, and how. Front. Public Health.

[125] De Jonge B.L.M., Karlowsky J.A., Kazmierczak K.M., Biedenbach D.J., Sahm D.F., Nichols W.W. (2016). In vitro susceptibility to ceftazidime-avibactam of carbapenem-nonsusceptible enterobacteriaceae isolates collected during the INFORM global surveillance study (2012 to 2014). Antimicrob. Agents Chemother..

[126] Cho J.C., Zmarlicka M.T., Shaeer K.M., Pardo J. (2018). Meropenem/Vaborbactam, the First Carbapenem/β-Lactamase Inhibitor Combination. Ann. Pharmacother..

[127] Pfaller M.A., Huband M.D., Mendes R.E., Flamm R.K., Castanheira M. (2018). In vitro activity of meropenem/vaborbactam and characterisation of carbapenem resistance mechanisms among carbapenem-resistant Enterobacteriaceae from the 2015 meropenem/vaborbactam surveillance programme. Int. J. Antimicrob. Agents.

[128] Campanella T.A., Gallagher J.C. (2020). A clinical review and critical evaluation of imipenem-relebactam: Evidence to date. Infect. Drug Resist..

[129] Lob S.H., Karlowsky J.A., Young K., Motyl M.R., Hawser S., Kothari N.D., Sahm D.F. (2020). In vitro activity of imipenem-relebactam against resistant phenotypes of Enterobacteriaceae and Pseudomonas aeruginosa isolated from intraabdominal and urinary tract infection samples-SMART Surveillance Europe 2015–2017. J. Med. Microbiol..

[130] Yang Q., Zhang H., Yu Y., Kong H., Duan Q., Wang Y., Zhang S., Sun Z., Liao K., Gu L. (2020). In vitro activity of imipenem/relebactam against enterobacteriaceae isolates obtained from intra-Abdominal, respiratory tract, and urinary tract infections in china: Study for monitoring antimicrobial resistance trends (smart), 2015–2018. Clin. Infect. Dis..

[131] Jean S.S., Lee W.S., Lam C., Hsu C.W., Chen R.J., Hsueh P.R. (2015). Carbapenemase-producing Gram-negative bacteria: Current epidemics, antimicrobial susceptibility and treatment options. Future Microbiol..

[132] Ract P., Compain F., Robin F., Decre D., Gallah S., Podglajen I. (2019). Synergistic in vitro activity between aztreonam and amoxicillin–clavulanate against Enterobacteriaceae-producing class B and/or class D carbapenemases with or without extended-spectrum β-lactamases. J. Med. Microbiol..

[133] Wenzler E., Deraedt M.F., Harrington A.T., Danizger L.H. (2017). Synergistic activity of ceftazidime-avibactam and aztreonam against serine and metallo-β-lactamase-producing gram-negative pathogens. Diagn Microbiol. Infect. Dis..

[134] Maraki S., Mavromanolaki V.E., Moraitis P., Stafylaki D., Kasimati A., Magkafouraki E., Scoulica E. (2021). Ceftazidime-avibactam, meropenen-vaborbactam, and imipenem-relebactam in combination with aztreonam against multidrug-resistant, metallo-β-lactamase-producing Klebsiella pneumoniae. Eur. J. Clin. Microbiol. Infect. Dis..

[135] Mushtaq S., Sadouki Z., Vickers A., Livermore D.M., Woodford N. (2020). In Vitro Activity of Cefiderocol, a Siderophore Cephalosporin, against Multidrug-Resistant Gram-Negative Bacteria. Antimicrob. Agents Chemother..

[136] Kohira N., Hackel M.A., Ishioka Y., Kuroiwa M., Sahm D.F., Sato T., Maki H., Yamano Y. (2020). Reduced susceptibility mechanism to cefiderocol, a siderophore cephalosporin, among clinical isolates from a global surveillance programme (SIDERO-WT-2014). J. Glob. Antimicrob. Resist..

[137] Satlin M.J. (2021). Languid Uptake of Ceftazidime-Avibactam for Carbapenem-Resistant Gram-Negative Infections and Continued Reliance on Polymyxins. Clin. Infect. Dis..

[138] Bassetti M., Echols R., Matsunaga Y., Ariyasu M., Doi Y., Ferrer R., Lodise T.P., Naas T., Niki Y., Paterson D.L. (2021). Efficacy and safety of cefiderocol or best available therapy for the treatment of serious infections caused by carbapenem-resistant Gram-negative bacteria (CREDIBLE-CR): A randomised, open-label, multicentre, pathogen-focused, descriptive, phase 3 trial. Lancet Infect. Dis..

[139] Cardozo C., Rico V., Agüero D., Soriano A. (2019). Antibiotic selection in the treatment of acute invasive infections by Pseudomonas aeruginosa. Rev. Esp. Quimioter..

[140] Torres A., Niederman M.S., Chastre J., Ewig S., Fernandez-Vandellos P., Hanberger H., Kollef M., Bassi G.L., Luna C.M., Martin-Loeches I. (2017). International ERS/ESICM/ESCMID/ALAT guidelines for the management of hospital-acquired pneumonia and ventilator-associated pneumonia. Eur. Respir. J..

[141] Klastersky J., de Naurois J., Rolston K., Rapoport B., Maschmeyer G., Aapro M., Herrstedt J., on behalf of the ESMO Guidelines Committee Management of febrile neutropaenia (2016). ESMO clinical practice guidelines. Ann. Oncol..

[142] Lodise T.P., Patel N., Kwa A., Graves J., Furuno J.P., Graffunder E., Lomaestro B., McGregor J.C. (2007). Predictors of 30-day mortality among patients with Pseudomonas aeruginosa bloodstream infections: Impact of delayed appropriate antibiotic selection. Antimicrob. Agents Chemother..

[143] Onorato L., Macera M., Calò F., Cirillo P., Di Caprio G., Coppola N. (2022). Beta-lactam monotherapy or combination therapy for bloodstream infections or pneumonia due to Pseudomonas aeruginosa: A meta-analysis. Int. J. Antimicrob. Agents.

[144] Mogyoródi B., Csékó A.B., Hermann C., Gál J., Iványi Z.D. (2022). Ceftolozane/tazobactam versus colistin in the treatment of ventilator-associated pneumonia due to extensively drug-resistant Pseudomonas aeruginosa. Sci. Rep..

[145] Halat D.H., Moubareck C.A. (2020). The current burden of carbapenemases: Review of significant properties and dissemination among gram-negative bacteria. Antibiotics.

[146] Karakonstantis S., Kritsotakis E.I., Gikas A. (2020). Treatment options for K. pneumoniae, P. aeruginosa and A. baumannii co-resistant to carbapenems, aminoglycosides, polymyxins and tigecycline: An approach based on the mechanisms of resistance to carbapenems. Infection.

[147] Lemos E.V., de la Hoz F.P., Einarson T.R., Mcghan W.F., Quevedo E., Castañeda C., Kawai K. (2014). Carbapenem resistance and mortality in patients with Acinetobacter baumannii infection: Systematic review and meta-analysis. Clin. Microbiol. Infect..

[148] Wagenlehner F., Lucenteforte E., Pea F., Soriano A., Tavoschi L., Steele V.R., Henriksen A.S., Longshaw C., Manissero D., Pecini R. (2021). Systematic review on estimated rates of nephrotoxicity and neurotoxicity in patients treated with polymyxins. Clin. Microbiol. Infect..

[149] Zheng J.Y., Huang S.S., Huang S.H., Ye J.J. (2020). Colistin for pneumonia involving multidrug-resistant Acinetobacter calcoaceticus-Acinetobacter baumannii complex. J. Microbiol. Immunol. Infect..

[150] Paul M., Daikos G.L., Durante-Mangoni E., Yahav D., Carmeli Y., Benattar Y.D., Skiada A., Andini R., Eliakim-Raz N., Nutman A. (2018). Colistin alone versus colistin plus meropenem for treatment of severe infections caused by carbapenem-resistant Gram-negative bacteria: An open-label, randomised controlled trial. Lancet Infect. Dis..

[151] Huang C., Chen I., Tang T. (2022). Colistin Monotherapy versus Colistin plus Meropenem Combination Therapy for the Treatment of Multidrug-Resistant Acinetobacter baumannii Infection: A Meta-Analysis. J. Clin. Med..

[152] Kengkla K., Kongpakwattana K., Saokaew S., Apisarnthanarak A., Chaiyakunapruk N. (2018). Comparative efficacy and safety of treatment options for MDR and XDR Acinetobacter baumannii infections: A systematic review and network meta-analysis. J. Antimicrob. Chemother..

[153] Zalts R., Neuberger A., Hussein K., Raz-Pasteur A., Geffen Y., Mashiach T., Finkelstein R. (2016). Treatment of Carbapenem-Resistant Acinetobacter baumannii Ventilator-Associated Pneumonia: Retrospective Comparison between Intravenous Colistin and Intravenous Ampicillin Sulbactam. Am. J. Ther..

[154] Liu J., Shu Y., Zhu F., Feng B., Zhang Z., Liu L., Wang G. (2021). Comparative efficacy and safety of combination therapy with high-dose sulbactam or colistin with additional antibacterial agents for multiple drug-resistant and extensively drug-resistant Acinetobacter baumannii infections: A systematic review and network meta-analysis. J. Glob. Antimicrob. Resist..

[155] Beganovic M., Daffinee K.E., Luther M.K., LaPlante K.L. (2021). Minocycline alone and in combination with polymyxin b, meropenem, and sulbactam against carbapenem-susceptible and -resistant acinetobacter baumannii in an in vitro pharmacodynamic model. Antimicrob. Agents Chemother..

[156] Russo A., Bassetti M., Bellelli V., Bianchi L., Marincola Cattaneo F., Mazzocchetti S., Paciacconi E., Cottini F., Schiattarella A., Tufaro G. (2021). Efficacy of a Fosfomycin-Containing Regimen for Treatment of Severe Pneumonia Caused by Multidrug-Resistant Acinetobacter baumannii: A Prospective, Observational Study. Infect. Dis. Ther..

[157] Jung S.Y., Lee S.H., Lee S.Y., Yang S., Noh H., Chung E.K., Lee J.I. (2017). Antimicrobials for the treatment of drug-resistant Acinetobacter baumannii pneumonia in critically ill patients: A systemic review and Bayesian network meta-analysis. Crit. Care.

[158] Sirijatuphat R., Thamlikitkul V. (2014). Preliminary study of colistin versus colistin plus fosfomycin for treatment of carbapenem-resistant Acinetobacter baumannii infections. Antimicrob. Agents Chemother..

[159] Santimaleeworagun W., Wongpoowarak P., Chayakul P., Pattharachayakul S., Tansakul P., Garey K.W. (2011). In vitro activity of colistin or sulbactam in combination with fosfomycin or imipenem against clinical isolates of carbapenem-resistant acinetobacter baumannii producing OXA-23 carbapenemases. Southeast Asian J. Trop. Med. Public Health.

[160] Wei W., Yang H., Liu Y., Ye Y., Li J. (2016). In vitro synergy of colistin combinations against extensively drug-resistant Acinetobacter baumannii producing OXA-23 carbapenemase. J. Chemother..

[161] Portsmouth S., van Veenhuyzen D., Echols R., Machida M., Ferreira J.C.A., Ariyasu M., Tenke P., Nagata T. (2018). Den. Cefiderocol versus imipenem-cilastatin for the treatment of complicated urinary tract infections caused by Gram-negative uropathogens: A phase 2, randomised, double-blind, non-inferiority trial. Lancet Infect. Dis..

[162] Wunderink R.G., Matsunaga Y., Ariyasu M., Clevenbergh P., Echols R., Kaye K.S., Kollef M., Menon A., Pogue J.M., Shorr A.F. (2021). Cefiderocol versus high-dose, extended-infusion meropenem for the treatment of Gram-negative nosocomial pneumonia (APEKS-NP): A randomised, double-blind, phase 3, non-inferiority trial. Lancet Infect. Dis..

[163] Falcone M., Tiseo G., Leonildi A., Della Sala L., Vecchione A., Barnini S., Farcomeni A., Menichetti F. (2022). Cefiderocol-Compared to Colistin-Based Regimens for the Treatment of Severe Infections Caused by Carbapenem-Resistant *Acinetobacter baumannii*. Antimicrob. Agents Chemother..

[164] Pascale R., Pasquini Z., Bartoletti M., Caiazzo L., Fornaro G., Bussini L., Volpato F., Marchionni E., Rinaldi M., Trapani F. (2021). Cefiderocol treatment for carbapenem-resistant *Acinetobacter baumannii* infection in the ICU during the COVID-19 pandemic: A multicentre cohort study. JAC Antimicrob. Resist..

[165] Seifert H., Stefanik D., Olesky M., Higgins P.G. (2020). In vitro activity of the novel fluorocycline TP-6076 against carbapenem-resistant *Acinetobacter baumannii*. Int. J. Antimicrob. Agents.

[166] Morrissey I., Olesky M., Hawser S., Lob S.H., Karlowsky J.A., Corey G.R., Bassetti M., Fyfe C. (2020). In vitro activity of eravacycline against Gram-negative bacilli isolated in clinical laboratories worldwide from 2013 to 2017. Antimicrob. Agents Chemother..

[167] Solomkin J., Evans D., Slepavicius A., Lee P., Marsh A., Tsai L., Sutcliffe J.A., Horn P. (2017). Assessing the efficacy and safety of Eravacycline vs Ertapenem in complicated intra-abdominal infections in the Investigating Gram-Negative Infections Treated with Eravacycline (IGNITE 1) trial a randomized clinical trial. JAMA Surg..

[168] Solomkin J.S., Gardovskis J., Lawrence K., Montravers P., Sway A., Evans D., Tsai L. (2019). IGNITE4: Results of a phase 3, randomized, multicenter, prospective trial of eravacycline vs meropenem in the treatment of complicated intraabdominal infections. Clin. Infect. Dis..

[169] Chang Y.T., Lin C.Y., Chen Y.H., Hsueh P.R. (2015). Update on infections caused by Stenotrophomonas maltophilia with particular attention to resistance mechanisms and therapeutic options. Front. Microbiol..

[170] Gales A.C., Seifert H., Gur D., Castanheira M., Jones R.N., Sader H.S. (2019). Antimicrobial Susceptibility of acinetobacter calcoaceticus-acinetobacter baumannii complex and stenotrophomonas maltophilia clinical isolates: Results from the SENTRY Antimicrobial Surveillance Program (1997–2016). Open Forum. Infect. Dis..

[171] Junco S.J., Bowman M.C., Turner R.B. (2021). Clinical outcomes of Stenotrophomonas maltophilia infection treated with trimethoprim/sulfamethoxazole, minocycline, or fluoroquinolone monotherapy. Int. J. Antimicrob. Agents.

[172] Ko J.H., Kang C.I., Cornejo-Juárez P., Yeh K.M., Wang C.H., Cho S.Y., Gözel M.G., Kim S.H., Hsueh P.R., Sekiya N. (2019). Fluoroquinolones versus trimethoprim-sulfamethoxazole for the treatment of Stenotrophomonas maltophilia infections: A systematic review and meta-analysis. Clin. Microbiol. Infect..

[173] Cho S.Y., Kang C.I., Kim J., Ha Y.E., Chung D.R., Lee N.Y., Peck K.R., Song J.H. (2014). Can levofloxacin be a useful alternative to trimethoprim-Sulfamethoxazole for treating stenotrophomonas maltophilia bacteremia?. Antimicrob. Agents Chemother..

[174] Flamm R.K., Shortridge D., Castanheira M., Sader H.S., Pfaller M.A. (2019). In vitro activity of minocycline against U.S. Isolates of *Acinetobacter baumannii*-*Acinetobacter calcoaceticus* Species Complex, *Stenotrophomonas maltophilia*, and *Burkholderia cepacia* Complex: Results from the SENTRY antimicrobial surveillance program, 2014 to 2018. Antimicrob. Agents Chemothe..

[175] Biagi M., Vialichka A., Jurkovic M., Wu T., Shajee A., Lee M., Patel S., Mendes R.E., Wenzler E. (2020). Activity of cefiderocol alone and in combination with levofloxacin, minocycline, polymyxin B, or trimethoprim-sulfamethoxazole against multidrug-resistant *Stenotrophomonas maltophilia*. Antimicrob. Agents Chemother..

[176] Hsueh S.C., Lee Y.J., Huang Y.T., Liao C.H., Tsuji M., Hsueh P.R. (2019). In vitro activities of cefiderocol, ceftolozane/tazobactam, ceftazidime/avibactam and other comparative drugs against imipenem-resistant Pseudomonas aeruginosa and Acinetobacter baumannii, and *Stenotrophomonas maltophilia*, all associated with bloodstream infections in Taiwan. J. Antimicrob. Chemother..

[177] Stracquadanio S., Torti E., Longshaw C., Henriksen A.S., Stefani S. (2021). In vitro activity of cefiderocol and comparators against isolates of Gram-negative pathogens from a range of infection sources: SIDERO-WT-2014–2018 studies in Italy. J. Glob. Antimicrob. Resist..

[178] Mojica M.F., Papp-Wallace K.M., Taracila M.A., Barnes M.D., Rutter J.D., Jacobs M.R., LiPuma J.J., Walsh T.J., Vila A.J., Bonomo R.A. (2017). Avibactam restores the susceptibility of clinical isolates of *Stenotrophomonas maltophilia* to aztreonam. Antimicrob. Agents Chemother..

[179] Lin Q., Zou H., Chen X., Wu M., Ma D., Yu H., Niu S., Huang S. (2021). Avibactam potentiated the activity of both ceftazidime and aztreonam against S. maltophilia clinical isolates in vitro. BMC Microbiol..

[180] Mauri C., Maraolo A.E., Di Bella S., Luzzaro F., Principe L. (2021). The revival of aztreonam in combination with avibactam against metallo-β-lactamase-producing gram-negatives: A systematic review of in vitro studies and clinical cases. Antibiotics.

[181] Sader H.S., Duncan L.R., Arends S.J.R., Carvalhaes C.G., Castanheira M. (2020). Antimicrobial Activity of Aztreonam-Avibactam and Comparator Agents When Tested against a Large Collection of Contemporary Stenotrophomonas maltophilia Isolates from Medical Centers Worldwide. Antimicrob. Agents Chemother..

[182] Mojica M.F., Ouellette C.P., Leber A., Becknell M.B., Ardura M.I., Perez F., Shimamura M., Bonomo R.A., Aitken S.L., Shelburne S.A. (2016). Successful treatment of bloodstream infection due to metallo-β-lactamase-producing Stenotrophomonas maltophilia in a renal transplant patient. Antimicrob. Agents Chemother..

[183] Diarra A., Pascal L., Carpentier B., Baclet N., Cabaret P., Georgel A.F., Dubreuil L., Weyrich P. (2021). Successful use of avibactam and aztreonam combination for a multiresistant Stenotrophomonas maltophilia bloodstream infection in a patient with idiopathic medullary aplasia. Infect. Dis. Now..

[184] Horcajada J.P., Montero M., Oliver A., Sorlí L., Luque S., Gómez-Zorrilla S., Benito N., Grau S. (2019). Epidemiology and treatment of multidrug-resistant and extensively drug-resistant Pseudomonas aeruginosa infections. Clin. Microbiol. Rev..

[185] Hu Y., Li L., Li W., Xu H., He P., Yan X., Dai H. (2013). Combination antibiotic therapy versus monotherapy for Pseudomonas aeruginosa bacteraemia: A meta-analysis of retrospective and prospective studies. Int. J. Antimicrob. Agents.

[186] Vardakas K.Z., Tansarli G.S., Bliziotis I.A., Falagas M.E. (2013). β-Lactam plus aminoglycoside or fluoroquinolone combination versus β-lactam monotherapy for Pseudomonas aeruginosa infections: A meta-analysis. Int. J. Antimicrob. Agents.

[187] Tang S.Y., Zhang S.W., Wu J.D., Wu F., Zhang J., Dong J.T., Guo P., Zhang D.L., Yang J.T., Zhang W.J. (2018). Comparison of mono- and combination antibiotic therapy for the treatment of Pseudomonas aeruginosa bacteraemia: A cumulative meta-analysis of cohort studies. Exp. Ther. Med..

[188] Wang J., Niu H., Wang R., Cai Y. (2019). Safety and efficacy of colistin alone or in combination in adults with Acinetobacter baumannii infection: A systematic review and meta-analysis. Int. J. Antimicrob. Agents.

[189] Makris D., Petinaki E., Tsolaki V., Manoulakas E., Mantzarlis K., Apostolopoulou O., Sfyras D., Zakynthinos E. (2018). Colistin versus colistin combined with ampicillin-sulbactam for multiresistant Acinetobacter baumannii ventilator-associated pneumonia treatment: An open-label prospective study. Indian J. Crit. Care Med..

[190] Novelli A., Del Giacomo P., Rossolini G.M., Tumbarello M. (2020). Meropenem/vaborbactam: A next generation β-lactam β-lactamase inhibitor combination. Expert Rev. Anti. Infect. Ther..

[191] Tamma P.D., Aitken S.L., Bonomo R.A., Mathers A.J., van Duin D., Clancy C.J. (2021). Infectious Diseases Society of America Guidance on the Treatment of AmpC β-Lactamase–Producing Enterobacterales, Carbapenem-Resistant Acinetobacter baumannii, and Stenotrophomonas maltophilia Infections. Clin. Infect. Dis..

[192] Falcone M., Daikos G.L., Tiseo G., Bassoulis D., Giordano C., Galfo V., Leonildi A., Tagliaferri E., Barnini S., Sani S. (2021). Efficacy of Ceftazidime-avibactam plus Aztreonam in Patients with Bloodstream Infections Caused by Metallo-β-lactamase-Producing Enterobacterales. Clin. Infect. Dis..

[193] Sousa A., Pérez-Rodríguez M.T., Soto A., Rodríguez L., Pérez-Landeiro A., Martínez-Lamas L., Nodar A., Crespo M. (2018). Effectiveness of ceftazidime/avibactam as salvage therapy for treatment of infections due to OXA-48 carbapenemase-producing Enterobacteriaceae. J. Antimicrob. Chemother..

